# The architecture of redox microdomains: Cascading gradients and peroxiredoxins’ redox-oligomeric coupling integrate redox signaling and antioxidant protection

**DOI:** 10.1016/j.redox.2023.103000

**Published:** 2023-12-21

**Authors:** Matthew Griffith, Adérito Araújo, Rui Travasso, Armindo Salvador

**Affiliations:** a CNC - Centre for Neuroscience Cell Biology, University of Coimbra, UC-Biotech, Parque Tecnológico de Cantanhede, Núcleo 4, Lote 8, 3060-197, Cantanhede, Portugal; b Department of Mathematical Sciences, University of Bath, Claverton Down, Bath, BA2 7AY, UK; c CMUC, Department of Mathematics, University of Coimbra, Largo D. Dinis, 3004-143, Coimbra, Portugal; d CFisUC, Department of Physics, University of Coimbra, Coimbra, Rua Larga, 3004-516, Coimbra, Portugal; e Coimbra Chemistry Center ‐ Institute of Molecular Sciences (CQC‐IMS), University of Coimbra, Rua Larga, 3004-535, Coimbra, Portugal; f Institute for Interdisciplinary Research, University of Coimbra, Casa Costa Alemão, Rua Dom Francisco de Lemos, 3030-789, Coimbra, Portugal

**Keywords:** Redox microdomains, Hydrogen peroxide, Peroxiredoxins, Redox signaling, Reaction-diffusion model, Total protein concentration gradients

## Abstract

In the cytosol of human cells under low oxidative loads, hydrogen peroxide is confined to microdomains around its supply sites, due to its fast consumption by peroxiredoxins. So are the sulfenic and disulfide forms of the 2-Cys peroxiredoxins, according to a previous theoretical analysis [Travasso et al.*, Redox Biology* 15 (2017) 297]. Here, an extended reaction-diffusion model that for the first time considers the differential properties of human peroxiredoxins 1 and 2 and the thioredoxin redox cycle predicts important new aspects of the dynamics of redox microdomains. The peroxiredoxin 1 sulfenates and disulfides are more localized than the corresponding peroxiredoxin 2 forms, due to the former peroxiredoxin's faster resolution step. The thioredoxin disulfides are also localized. As the H_2_O_2_ supply rate (*v*_sup_) approaches and then surpasses the maximal rate of the thioredoxin/thioredoxin reductase system (*V*), these concentration gradients become shallower, and then vanish. At low *v*_sup_ the peroxiredoxin concentration determines the H_2_O_2_ concentrations and gradient length scale, but as *v*_sup_ approaches *V*, the thioredoxin reductase activity gains influence. A differential mobility of peroxiredoxin disulfide dimers *vs.* reduced decamers enhances the redox polarity of the cytosol: as *v*_sup_ approaches *V*, reduced decamers are preferentially retained far from H_2_O_2_ sources, attenuating the local H_2_O_2_ buildup. Substantial total protein concentration gradients of both peroxiredoxins emerge under these conditions, and the concentration of reduced peroxiredoxin 1 far from the H_2_O_2_ sources even increases with *v*_sup_. Altogether, the properties of 2-Cys peroxiredoxins and thioredoxin are such that localized H_2_O_2_ supply induces a redox and functional polarization between source-proximal regions (redox microdomains) that facilitate peroxiredoxin-mediated signaling and distal regions that maximize antioxidant protection.

## Highlights

• First model of spatio-temporal dynamics of Prdx1,2/Trx1/TrxR system in human cells.
• Prdx1,2 sulfenates and disulfides and Trx1 disulfides are localized within 2 μm from H_2_O_2_ sources.
• Prdx1 sulfenates and disulfides are more strongly localized than Prdx2's.
• Prdx1/2 decamer dissociation upon oxidation protects regions far from H_2_O_2_ sources.
• Prdx1/2's properties promote redox and functional polarization of the cytosol.

## Graphical abstract

**Figure:**
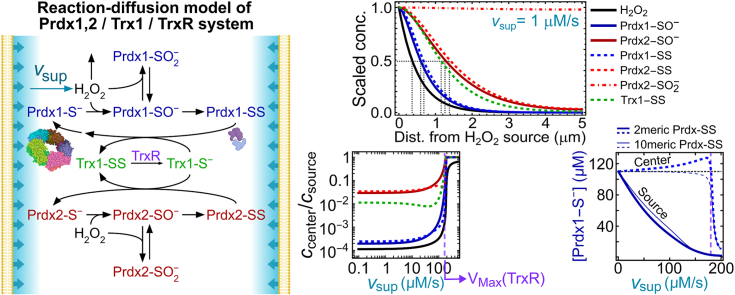

## Abbreviations

ASK1: Apoptosis signal-regulating kinase 1
Prdx1: peroxiredoxin 1
Prdx2: Peroxiredoxin 2
SOD1: superoxide dismutase 1
Srx: Sulfiredoxin
Trx1: Thioredoxin 1
TrxR: Thioredoxin reductase

## 1 Introduction

H_2_O_2_-mediated redox signaling occurs primarily through the oxidation of protein thiols [1]. However, most protein thiol targets that are rapidly oxidized upon cell stimulation are poorly H_2_O_2_-reactive (*k* = 1-200 M^−1^s^−1^ [[2], [3], [4], [5]]), which precludes significant direct oxidation by H_2_O_2_ in the observed timescale under physiological conditions [6]. How such targets can be oxidized in the observed signaling time frames at nM [7,8] cytosolic H_2_O_2_ concentrations, and how such a simple molecule as H_2_O_2_ can warrant specific signaling are key questions in Redox Biology. Among the mechanisms proposed to explain these questions [[9], [10], [11], [12], [13], [14], [15], [16], [17], [18], [19]], redox relays where peroxiredoxins (Prdx) act as the H_2_O_2_ receptors that in turn oxidize the redox targets are gaining prominence.

**Fig. 1:**
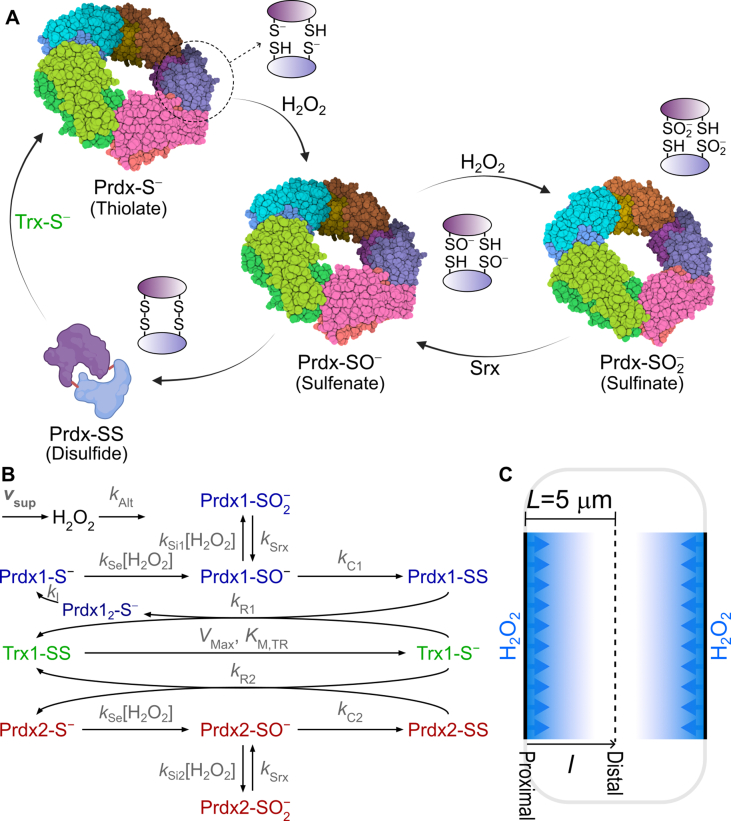
(A) Redox cycle of 2-Cys peroxiredoxins and its coupling to oligomeric states. Monomers associated in the same dimer are indicated by different shades of the same color. The dashed circle points to a diagram of the two active sites' redox state in a dimer. Dimers whose active sites are converted to the disulfide form, tend to dissociate from the decamers. In turn, decamers in the sulfinic form may associate into even higher order structures, which are not considered in the present model (prepared with the help of BioRender). (B) Modeled reaction scheme. The kinetic parameters for each process are indicated near the respective arrows. In the case of bimolecular reactions, the product of the rate constant by the co-reactant concentration are shown. Parameter meanings and their reference values are indicated in Table 1 (C) Modeled geometry. H_2_O_2_ is uniformly supplied over each membrane (thick black lines). The dashed line marks an axis of symmetry. The simulated domain corresponds to the region between the left-hand side membrane and the axis of symmetry. (For interpretation of the references to color in this figure legend, the reader is referred to the Web version of this article.)

Peroxiredoxins are legitimate H_2_O_2_ receptors because: (i) owing to their high reactivity (k ≈ 10^7^-10^8^ M^−1^s^−1^ [[20], [21], [22], [23]]) and abundance (>50 μM [24]) they capture nearly all the H_2_O_2_ supplied to their host cellular compartments at physiological rates; and (ii) they can specifically transmit this signal to a subset of proteins defined, e.g., by topological and electrostatic complementarity. The most abundant peroxiredoxins in the cytosol of human cells are peroxiredoxins 1 and 2 (Prdx1, Prdx2) [24,25]. These are 2-Cys peroxiredoxins, whose functional units are homodimers with monomers in an antiparallel conformation, each monomer containing two catalytically active Cys (Fig. 1A). One of these two Cys, the peroxidatic Cys (C_P_), is very rapidly oxidized by H_2_O_2_ to a sulfenate (Prdx-SO^−^). The sulfenate condenses with the other Cys (resolving Cys, C_R_) in the opposing monomer, forming a disulfide (Prdx-SS), in what is called the “resolution step”. It can also be oxidized to a sulfinate (Prdx-SO_2_^−^) by another H_2_O_2_ molecule (“hyperoxidation”). Whilst the disulfide can be readily reduced to the initial thiolate (Prdx-S^−^) by thioredoxin 1 (Trx1-S^−^), the sulfinate is slowly reduced to a sulfenate in an ATP- and GSH-dependent reaction catalyzed by sulfiredoxin (Srx). Trx1 that becomes oxidized to Trx1-SS in Prdx reduction is reduced by NADPH, under catalysis by Thioredoxin Reductase (TrxR).

**Table 1:** Processes considered and reference values of the corresponding parameters.

Reaction	Par.	Values	Refs.
→ H_2_O_2_	*v*_sup_	variable	See text
H_2_O_2_ →	*k*_Alt_	100 s^−1^	See text
Prdx1-S^−^ + H_2_O_2_ → Prdx1-SO^−^ + H_2_O	*k*_Se_	100 μM^−1^s^−1^	See text
Prdx2-S^−^ + H_2_O_2_ → Prdx2-SO^−^ + H_2_O	*k*_Se_	100 μM^−1^s^−1^	See text
Prdx1-SO^−^ → Prdx1-SS + H_2_O	*k*_C1_	11 s^−1^	[22,23,44]
Prdx2-SO^−^ → Prdx2-SS + H_2_O	*k*_C2_	0.5 s^−1^	[46]
Prdx1-SS + Trx1-S^−^ → Prdx1_2_-S^−^ + Trx1-SS	*k*_R1_	2.2 μM^−1^s^−1^	[45]
Prdx1_2_-S^−^ → Prdx1-S^−^	*k*_I_	15.8 s^−1^	[45]
Prdx2-SS + Trx1-S^−^ → Prdx2-S^−^ + Trx1-SS	*k*_R2_	0.61 μM^−1^s^−1^	[45]
Prdx1-SO^−^ + H_2_O_2_ → Prdx1-SO_2_^−^ + H_2_O	*k*_Si1_	0.0015 μM^−1^s^−1^	[11,24,44,47]
Prdx2-SO^−^ + H_2_O_2_ → Prdx2-SO_2_^−^ + H_2_O	*k*_Si2_	0.0034 μM^−1^s^−1^	[44,46,48]
Prdx1-SO_2_^−^ → Prdx1-SO^−^, Prdx2-SO_2_^−^ → Prdx2-SO^−^	*k*_Srx_	10^−3^ s^−1^	[24]
Trx1-SS → Trx1-S^−^	*V*_Max_	180 μM s^−1^	See text
	*K*_M_	1.8 μM	[49]
H_2_O_2_ diffusion	*D*_H_	3700 μm^2^s^-1^	[50]
Prdx1/2-S^−^, Prdx1/2-SO^−^, Prdx2-SO_2_^−^ diffusion	*D*_P10_	0.52 μm^2^s^-1^	See text
Prdx1/2-SS diffusion	*D*_P2_	0.9 μm^2^s^-1^	See text
Trx1-S^−^, Trx1-SS diffusion	*D*_Trx_	35 μm^2^s^-1^	See text
**Independent variables**			
Total Prdx1 concentration	Prdx1_T_	110 μM	[24]
Total Prdx2 concentration	Prdx2_T_	30 μM	[24]
Total Trx1 concentration	Trx1_T_	20 μM	[24]

The redox state of Prdx1 and Prdx2 influences their oligomeric state (Fig. 1A). The reduced forms are donut-shaped pentamers of dimers, and these decamers may associate in even higher order structures when hyperoxidized [[26], [27], [28], [29]]. In turn, the disulfide forms dissociate at least partially into dimers [[26], [27], [28], [29]]. Prdx1-SS decamers can be stabilized through the formation of dimer-to-dimer disulfide bridges via the oxidation of Cys83 under oxidative stress [30], and destabilized by glutathionylation of Cys83 [28]. Despite these complexities, studies with a homo-FRET probe suggest that in cells both Prdx1 and Prdx2 substantially dissociate into dimers when oxidized to their disulfide forms [31]. Both the large size and the extended hydrodynamic radius (relative to a compact globular shape) of the high molecular weight forms should substantially hinder their mobility in the cytosol relative to that of the dimers. However, the functional significance of these drastic changes in quaternary structure remains unclear.

Both Prdx-SO^−^ and Prdx-SS may in principle react with the thiols of target proteins, relaying the oxidizing equivalents. A recent screen [32] found a relatively low overlap between the binders of Prdx1 and Prdx2 in human cells, and distinct preferences for oxidizing their interactors via heterocondensation with Prdx-SO^−^ *vs*. thiol-disulfide exchange with Prdx-SS. This indicates that signaling through Prdx-mediated redox relays is Prdx-specific and prompts an examination of the underpinnings and functional implications of this specificity. A differential localization of the oxidized forms of these two Prdx may contribute to their distinct specificity. At the low H_2_O_2_ supply rates (*v*_sup_) prevailing under basal physiological conditions, the activity of the cytosolic 2-Cys Prdx limit H_2_O_2_'s concentration to the sub-nM to low-nM range and half-life to <1 ms [7,33]. A H_2_O_2_ molecule can diffuse over a range of just ≈0.5 μm within this short half-life. For this reason, under most physiological conditions cytosolic H_2_O_2_ is limited to sub-μm-scale domains (“microdomains”) around the supply sites. The latter inference from theoretical considerations [6,[34], [35], [36]], was confirmed by experiments using sensitive genetically encoded probes [8,[37], [38], [39], [40]], as recently reviewed [41]. At these low H_2_O_2_ supply rates, the oxidized forms of Prdx1 and Prdx2 are also circumscribed to microdomains [6]. This is because, although these peroxiredoxin forms are much longer lived than H_2_O_2_, their diffusion in the cytosol is also much slower due to their large size.

In ref. [42] we examined the spatial distribution of H_2_O_2_ and Prdx redox forms using a simple reaction-diffusion model that considered a single 2-Cys peroxiredoxin and neglected the redox cycle of Trx1. The experimental determination of the rate constants for all the main steps in the catalytic cycles of Prdx1 and Prdx2 has been accomplished more recently as result of the work of several groups [[20], [21], [22], [23],^29^,[43], [44], [45]]. This information has allowed us to set up the first reaction-diffusion model of the peroxiredoxin-thioredoxin system in the cytosol of human cells that accounts for the differential properties of these Prdx and for the Trx1 redox cycle. Using this model, we examined several questions prompted by the discussion above that were beyond the scope of the previous [42] model: How do the concentration gradients of distinct Prdx species differ? Is oxidized Trx1 also localized? What determines the extent of localization of the various species and the collapse of the gradients? How does the effect of redox state on oligomerization influence the operation of the Prdx – Trx system?

The results reveal steep Prdx-SO^−^, Prdx-SS and Trx1-SS concentration gradients, substantially steeper for the Prdx1 than for the Prdx2 species. The gradients become shallower and then collapse as the H_2_O_2_ supply rate approaches and then surpasses the maximal rate of Prdx-SS reduction by the Trx1/TrxR system. In contrast to the results in Ref. [42], but consistent with a subsequent analysis based on a simpler model [24], this transition is not hysteretic (*i.e.*, trigger-like). Surprisingly, far from H_2_O_2_ sources the concentration of reduced Prdx1 even increases with increasing H_2_O_2_ supply rate, as a consequence of the coupling between peroxiredoxin's redox and oligomerization states. We discuss the implications of these results for redox signaling and antioxidant protection, and how they help explaining recent experimental observations.

## 2 Models, parameter estimates and methods

### 2.1 Model and parameter estimates

The considered reaction network is represented in Fig. 1B, and the corresponding parameters are shown in Table 1. The main considerations underlying the choice of geometry, reactions and parameter values are explained below.

*Geometry*. We consider a 10 µm-thick (except where otherwise noted) cell layer with a symmetry axis at the center, delimited by a membrane on each side (Fig. 1C). This geometry allows the spatial distribution of the various chemical species to be modeled considering a single spatial dimension. Regions adjacent to the H_2_O_2_ sources (*i.e.*, to the membranes) will be denoted as (source-)“proximal”, whereas the regions farthest from the sources (*i.e.*, at the center of the considered spatial domain) will be denoted as “distal” (Fig. 1C).

*H*_*2*_*O*_*2*_*supply* is assumed to occur equally at both membranes, at a constant rate (*v*_sup_). H_2_O_2_ likely crosses membranes passively in some cell types and organelles [51], through channels such as peroxiporins in others [[52], [53], [54], [55], [56]], and may also be produced at the surface of some internal membranes. The model is agnostic about these alternative modes of H_2_O_2_ supply. Analysis of theoretical and experimental evidence in the literature [7,42,[57], [58], [59], [60], [61], [62], [63]] (Supplementary Information Section 1, SI1) suggests that basal *v*_sup_ in human cells are no higher than the low μM s^−1^. Higher values are likely during oxidative stress, although achieving 100s of uM s^−1^ may be unlikely.

*H*_*2*_*O*_*2*_*-clearing pathways* other than reaction with Prdx1 and Prdx2 were aggregated in a single first-order process with rate constant *k*_Alt_ = 100 s^−1^. This value is in the range of estimates that include the activities of peroxiredoxin 6, glutathione peroxidases, and catalase for a variety of human cell lines [24].

*Catalytic cycle of Prdx1 and Prdx2*. The core reaction network includes the catalytic cycles of both the major 2-Cys peroxiredoxins in the cytosol of human cells, according to an independent-sites model. A modest intra-dimer cooperativity of Prdx2 [46] and likely also of Prdx1, can be neglected without major impact on the conclusions of this work. The choice of values for the rate constants of each step is justified below.

Rate constants for the sulfenylation of Prdx1 and Prdx2 by H_2_O_2_ are in the range of 13–160 μM^−1^s^−1^ [[20], [21], [22], [23]], with recent determinations tending to similar higher values for both these peroxiredoxins. Therefore, we adopted the value 100 μM^−1^s^−1^ for both.

The resolution rate constant for Prdx1-SO^−^ is taken as the mean between quite similar values in recent literature: 12.9 s^−1^ [22], 9 s^−1^ [23], 11-12 s^−1^ [44]. For Prdx2-SO^−^, this was determined in Ref. [46], which reports that the formation of the first disulfide in a dimer approximately halves the resolution rate constant of the sulfenate at the second site. Because this work mainly addresses the events at low oxidative loads, we adopted the rate constant that pertains to dimers still devoid of disulfides. The adopted value is also in the range of other recent determinations: 0.25 s^−1^ [23], 0.64 s^−1^ [22], 0.2–0.3 s^−1^ [44].

The rate constants for Prdx1-SS and Prdx2-SS reduction by human Trx1 were recently determined as 2.2 μM^−1^s^−1^ and 0.61 μM^−1^s^−1^, respectively [45]. The kinetics of Prdx2-SS reduction by Trx1-S^−^ in the experiments in Ref. [45] was well fitted by a model treating this process as a simple bimolecular reaction. However, in the case of Prdx1 reduction the second-order step appears to be followed by a first-order step with rate constant 15.8 s^−1^ [45]. The molecular events associated to the latter step remain unclear. Here we consider that the second-order step yields peroxidase-inactive Prdx1-S^−^ *dimers* (Prdx1_2_-S^−^), which readily associate into peroxidase-competent decamers immediately after the first-order step. A recent reanalysis [64] of Isothermal Titration Calorimetry data from Ref. [65] supports such a fast association of Prdx1-S^−^ dimers into decamers. Results below show that neither the 1-step *vs*. 2-step Prdx1-SS reduction nor the oligomeric state of the Prdx1_2_-S^−^ species influence the main conclusions.

*Prdx1-SO*^*−*^*and Prdx2-SO*^*−*^*sulfinylation:* The adopted values for the rate constants of these processes are also averages of recent determinations: 0.00177 μM^−1^s^−1^ [44], 0.0013 μM^−1^s^−1^ [11,24,47] for Prdx1-SO^−^, 0.00197 μM^−1^s^−1^ [44], 0.0034 μM^−1^s^−1^ [46], 0.0042 μM^−1^s^−1^ [48] for Prdx2-SO^−^.

*Sulfiredoxin (Srx)-catalyzed reduction of peroxiredoxin sulfinic acids*: this is a complex GSH- and ATP-dependent process [[66], [67], [68]] whose kinetics remains incompletely characterized. The reduction of Prdx1-SO_2_^−^ in A549 cells follows first-order kinetics with *k*_Srx_ = 4.45 × 10^−3^ s^−1^ [24,69], and Prdx2-SO_2_^−^ tends to be more rapidly reduced than Prdx1-SO_2_^−^ in cells [70]. However, most cells examined in Ref. [24] contain substantially less Srx than A549 cells. So, we modeled Prdx1-SO_2_^−^ and Prdx2-SO_2_^−^ reduction as first-order processes with *k*_Srx_ = 10^−3^ s^−1^.

*Thioredoxin disulfide reduction* follows Henri-Michaelis-Menten kinetics. We assumed that the enzyme is saturated with NADPH, given its low *K*_M_(NADPH) = 6 μM [71]. The chosen *V*_Max_ is in the range of estimated activities for many human cell lines [24]. However, in aligning the results with the *v*_sup_ ranges discussed above one must keep in mind that some cells have *V*_Max_ values one order of magnitude lower [24].

*Diffusion*. For H_2_O_2_ we used the diffusion constant determined for a hydrogel with the approximate viscosity of the cytosol [50]. We estimated the diffusion constants for the peroxiredoxin decameric (*i.e.,* with the peroxidatic cysteines in thiolate, sulfenic and sulfinic forms) and dimeric (disulfide) forms from the experimentally determined diffusion constant for Immunoglobulin G in the cytosol [72] by applying the expression $D=D_{\text{IgG}}\sqrt[{3}]{{\text{MW}}_{\text{IgG}}/{\text{MW}}_{\text{Prdx}}}\text{,}$ with MW_Prdx_ replaced by the molecular weight of a Prdx decamer or of a dimer, respectively. The previous expression follows from the Stokes-Einstein relationship by assuming spherical proteins. The estimated diffusion constant for Trx1 is a mean of the values obtained by applying the expression above to the diffusion constant obtained for myoglobin diffusion in rat myocardium cells (MW_Myo_ = 17.7 kDa, *D*_Myo_ = 42.4 μm^2^s^-1^ [73]) and that obtained for 10 kDa Dextran in amphibian oocytes (*D*_Dex_ = 25 μm^2^s^-1^ [74]). These estimates are admittedly rough. However, because under the conditions of interest for this work the maximal concentrations and characteristic lengths of the concentration gradients scale approximately as *D*^−1/2^, the uncertainty in the diffusion constants has a limited effect on the results.

The reference values adopted for the total concentrations of Prdx1, Prdx2 and Trx1 (Table 1) are within the range and proportions of estimated values for HEK293 and other human cell lines [24].

### 2.2 Equations

The concentrations of the chemical species in the cytosol depend on time and distance from the membrane as mathematically described by the following system of reaction-diffusion partial differential equations, whose parameters are described in Table 1:

(1) $$\begin{array}{l} \frac{\partial \left[H_{2}O_{2}\right]}{\partial t}=D_{H}\frac{\partial^{2}\left[H_{2}O_{2}\right]}{\partial x^{2}}-(k_{\text{Alt}}+k_{\text{Se}}\left[\text{Prdx}1-S^{-}\right]+k_{\text{Si}1}\left[\text{Prdx}1-{\text{SO}}^{-}\right]+\\ +k_{\text{Se}}\left[\text{Prdx}2-S^{-}\right]+k_{\text{Si}2}\left[\text{Prdx}2-{\text{SO}}^{-}\right])\left[H_{2}O_{2}\right]\\ \frac{\partial \left[\text{Prdx}1-S^{-}\right]}{\partial t}=D_{P10}\frac{\partial^{2}\left[\text{Prdx}1-S^{-}\right]}{\partial x^{2}}+k_{I}\left[\text{Prdx}1_{2}-S^{-}\right]-k_{\text{Se}}\left[\text{Prdx}1-S^{-}\right]\left[H_{2}O_{2}\right]\\ \frac{\partial \left[\text{Prdx}1-{\text{SO}}^{-}\right]}{\partial t}=D_{P10}\frac{\partial^{2}\left[\text{Prdx}1-{\text{SO}}^{-}\right]}{\partial x^{2}}+k_{\text{Se}}\left[\text{Prdx}1-S^{-}\right]\left[H_{2}O_{2}\right]+k_{\text{Srx}}\left[\text{Prdx}1-{\text{SO}}_{2}^{-}\right]-k_{C1}\left[\text{Prdx}1-{\text{SO}}^{-}\right]-k_{\text{Si}1}\left[\text{Prdx}1-{\text{SO}}^{-}\right]\left[H_{2}O_{2}\right]\\ \frac{\partial \left[\text{Prdx}1-{\text{SO}}_{2}^{-}\right]}{\partial t}=D_{P10}\frac{\partial^{2}\left[\text{Prdx}1-{\text{SO}}_{2}^{-}\right]}{\partial x^{2}}+k_{\text{Si}1}\left[\text{Prdx}1-{\text{SO}}^{-}\right]\left[H_{2}O_{2}\right]-k_{\text{Srx}}\left[\text{Prdx}1-{\text{SO}}_{2}^{-}\right]\\ \frac{\partial \left[\text{Prdx}1-\text{SS}\right]}{\partial t}=D_{P2}\frac{\partial^{2}\left[\text{Prdx}1-\text{SS}\right]}{\partial x^{2}}+k_{C1}\left[\text{Prdx}1-{\text{SO}}^{-}\right]-k_{R1}\left[\text{Prdx}1-\text{SS}\right]\left[\text{Trx}1-S^{-}\right]\\ \frac{\partial \left[\text{Prdx}1_{2}-S^{-}\right]}{\partial t}=D_{P2}\frac{\partial^{2}\left[\text{Prdx}1_{2}-S^{-}\right]}{\partial x^{2}}+k_{R1}\left[\text{Prdx}1-\text{SS}\right]\left[\text{Trx}1-S^{-}\right]-k_{I}\left[\text{Prdx}1_{2}-S^{-}\right]\\ \frac{\partial \left[\text{Prdx}2-S^{-}\right]}{\partial t}=D_{P10}\frac{\partial^{2}\left[\text{Prdx}2-S^{-}\right]}{\partial x^{2}}+k_{R2}\left[\text{Prdx}2-\text{SS}\right]\left[\text{Trx}1-S^{-}\right]-k_{\text{Se}}\left[\text{Prdx}2-S^{-}\right]\left[H_{2}O_{2}\right]\\ \frac{\partial \left[\text{Prdx}2-{\text{SO}}^{-}\right]}{\partial t}=D_{P10}\frac{\partial^{2}\left[\text{Prdx}2-{\text{SO}}^{-}\right]}{\partial x^{2}}+k_{\text{Se}}\left[\text{Prdx}2-S^{-}\right]\left[H_{2}O_{2}\right]+k_{\text{Srx}}\left[\text{Prdx}2-{\text{SO}}_{2}^{-}\right]-k_{C2}\left[\text{Prdx}2-{\text{SO}}^{-}\right]-k_{\text{Si}2}\left[\text{Prdx}2-{\text{SO}}^{-}\right]\left[H_{2}O_{2}\right]\\ \frac{\partial \left[\text{Prdx}2-{\text{SO}}_{2}^{-}\right]}{\partial t}=D_{P10}\frac{\partial^{2}\left[\text{Prdx}2-{\text{SO}}_{2}^{-}\right]}{\partial x^{2}}+k_{\text{Si}2}\left[\text{Prdx}2-{\text{SO}}^{-}\right]\left[H_{2}O_{2}\right]-k_{\text{Srx}}\left[\text{Prdx}2-{\text{SO}}_{2}^{-}\right]\\ \frac{\partial \left[\text{Prdx}2-\text{SS}\right]}{\partial t}=D_{P2}\frac{\partial^{2}\left[\text{Prdx}2-\text{SS}\right]}{\partial x^{2}}+k_{C2}\left[\text{Prdx}2-{\text{SO}}^{-}\right]-k_{R2}\left[\text{Prdx}2-\text{SS}\right]\left[\text{Trx}1-S^{-}\right]\\ \frac{\partial \left[\text{Trx}1-S^{-}\right]}{\partial t}=D_{\text{Trx}}\frac{\partial^{2}\left[\text{Trx}1-S^{-}\right]}{\partial x^{2}}+\frac{V_{\mathrm{Max}}\left[\text{Trx}1-\text{SS}\right]}{K_{M}+\left[\text{Trx}1-\text{SS}\right]}-\left(k_{R1}\left[\text{Prdx}1-\text{SS}\right]+k_{R2}\left[\text{Prdx}2-\text{SS}\right]\right)\left[\text{Trx}1-S^{-}\right]\\ \frac{\partial \left[\text{Trx}1-\text{SS}\right]}{\partial t}=D_{\text{Trx}}\frac{\partial^{2}\left[\text{Trx}1-\text{SS}\right]}{\partial x^{2}}+\left(k_{R1}\left[\text{Prdx}1-\text{SS}\right]+k_{R2}\left[\text{Prdx}2-\text{SS}\right]\right)\left[\text{Trx}1-S^{-}\right]-\frac{V_{\mathrm{Max}}\left[\text{Trx}1-\text{SS}\right]}{K_{M}+\left[\text{Trx}1-\text{SS}\right]} \end{array}$$

All the concentrations have zero-flux boundary conditions except for that of H_2_O_2_ at the membrane side of the domain (*x* = 0). Here, the flux of H_2_O_2_ entering the domain per unit time is:

(2) $${\frac{\partial \left[H_{2}O_{2}\right]}{\partial x}|}_{x=0}=-\frac{L}{D_{H}}v_{\sup}$$

where *L* is the distance from the membranes to the cell center.

### 2.3 Methods

The equations were solved using the method of lines [75], in which a PDE is discretized in all but one dimension. Here we discretized the spatial dimension. This reduced the system to only one continuous dimension (time), allowing solutions to be computed via numerical integration techniques for ordinary differential equations (ODEs). We use a second-order central finite difference for the second order spatial derivative as well as a second-order central difference for the first order spatial derivative in the boundary conditions in order to preserve second-order accuracy in spatial derivatives across the whole system. A uniform mesh in space with size Δx = 0.1 μm is used.

To solve the resultant system of ODEs, we used the LSODA solver within the scipy package in Python [76]. This solver uses a combination of the Adams and BDF methods with automatic stiffness detection and switching between both methods. LSODA is a wrapper to the Fortran solver from ODEPACK [77]. It switches automatically between the non-stiff Adams method and the stiff BDF method with adaptive time stepping. The method was originally detailed in Ref. [78].

This integration method provided excellent total mass conservation in the system, with only a fractional loss (0.01 %) in the simulations with fastest concentration variations. The method of lines approach also allows the solver to adaptively change the time step as the simulation progresses. This means that small time steps are used initially when the system is subject to large changes, to preserve good mass conservation in the system, while larger time steps are used when the system approaches the steady state. We use this to obtain the results that follow in the next section.

## 3 Results

### 3.1 Dynamics of the concentrations after the onset of H_2_O_2_ supply

**Fig. 2:**
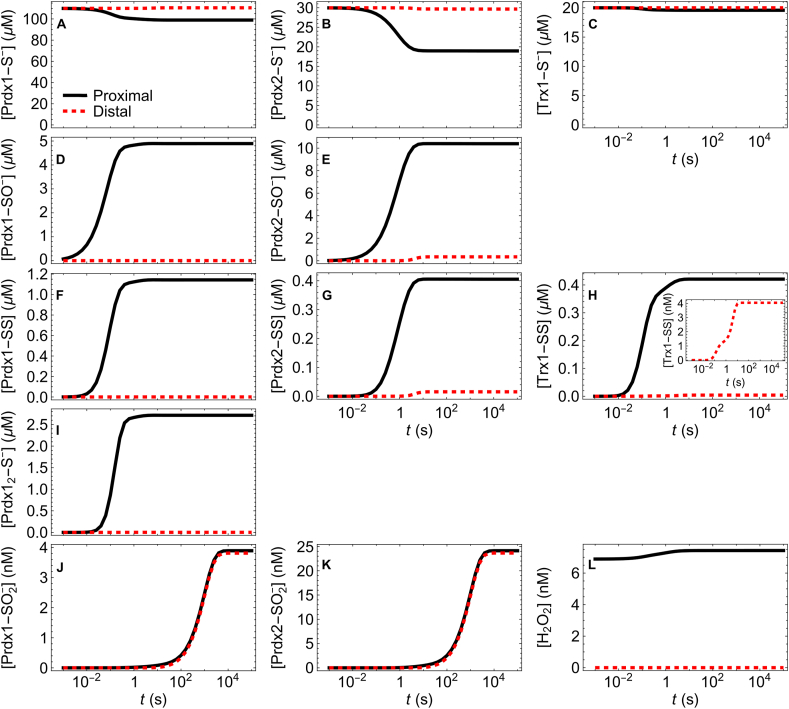
**Time course of the concentrations in the proximal (black) and distal (dashed red) regions after the onset of a 10 μM s**^**−**1^ **H**_**2**_**O**_**2**_**supply rate.** All the other parameters as in Table 1. Prdx1, Prdx2 and Trx1 are fully reduced at t = 0. The inset in panel H shows the time course of the concentration of Trx1-SS at the center of the cell. Note the logarithmic time scale. (For interpretation of the references to color in this figure legend, the reader is referred to the Web version of this article.)

We first examine the time course of the spatial distribution of the various species following the onset of a constant *v*_sup_ = 10 μM s^−1^ H_2_O_2_ supply rate, starting with the Prdx1, Prdx2 and Trx1 pools fully reduced. For this *v*_sup_ and the reference parameters in Table 1, there is a modest oxidation of these pools (Fig. 2A,B,C). The dynamics of the concentrations of H_2_O_2_ and of the oxidized forms of Prdx1, Prdx2 and Trx1 unfolds over a wide range of time scales, as follows. The H_2_O_2_ concentration gradient establishes and reaches a quasi-steady-state within 1 ms, as a consequence of the fast diffusion of this species and of the high concentrations and reactivity of Prdx1 and Prdx2 (Fig. 2L). The concentrations of Prdx1-SO^−^ and Prdx1-SS approach their quasi-steady-state in the 0.1 s time scale (Fig. 2D,F). In turn, the concentrations of the corresponding Prdx2 forms do so with some delay, in the seconds time scale, their gradients fully settling only by ≈10 s (Fig. 2E,G). The slower dynamics of these Prdx2 species is mainly due to this peroxiredoxin's lower resolution rate constant (*k*_C2_) relative to Prdx1's. The concentration of Trx1-SS settles in approximately the same time scale as Prdx1-SS in the proximal regions (Fig. 2H), but only by ≈10 s at the distal one (Fig. 2H inset). Finally, the concentrations of Prdx1-SO_2_^−^ and Prdx2-SO_2_^−^ take nearly 1 h to settle to a steady state, due to the slow Srx-catalyzed reduction of these species. This process is too slow to sustain significant Prdx-SO_2_^−^ concentration gradients (Fig. 2J and K). Nevertheless, the concentrations of Prdx1-SO_2_^−^ and Prdx2-SO_2_^−^ remain nM, because at this low *v*_sup_ sulfinylation of the sulfenic Prdx species cannot significantly compete with the condensation reaction.

### 3.2 At low oxidative loads Prdx1 disulfides and sulfenates are more localized than Prdx2's

**Fig. 3:**
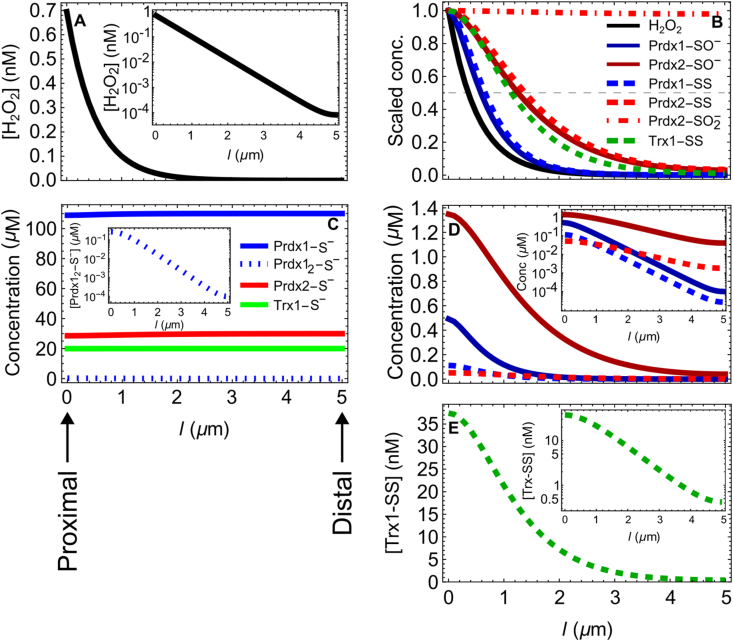
**Spatial concentration profiles at steady state for a constant low (1μMs^−1^) H**_**2**_**O**_**2**_**influx.** The membrane is located at l= 0 and the center of the cell at *l*= 5 μm. (A) H_2_O_2_ concentration. The inset shows the concentration in logarithmic scale. Note the exponential decay with the distance from the membrane. (B) Concentrations of the oxidized Prdx and Trx species scaled by their values adjacent to the membrane. The gray dashed horizontal line marks a 50 % decreased concentration relative to that at the proximal region. (C) Concentrations of the reduced Prdx and Trx species. The inset shows the distribution of the Prdx1_2_-S^−^ in logarithmic concentration coordinates. (D) Concentrations of the sulfenic and disulfide Prdx species. Color/dashing convention is the same as in (B). The inset shows the concentrations in logarithmic scale. Note the nearly exponential decrease with the distance from the membrane, the cross-over between the Prdx1-SS and Prdx2-SS concentrations, and the extremely low concentrations of the Prdx1 species towards the distal region. (E) Trx1-SS concentration. The inset shows the concentration in logarithmic scale. (For interpretation of the references to color in this figure legend, the reader is referred to the Web version of this article.)

The steady state spatial distributions of the various species deserve particular attention due to their relevance for redox signaling. For *v*_sup_ < 50 μM s^−1^, H_2_O_2_, the peroxiredoxins' sulfenic forms, as well as the peroxiredoxins' and Trx1's disulfide forms, are all localized near the H_2_O_2_ sources (Fig. 3A,B,D,E). Concretely, the concentrations of H_2_O_2_, Prdx1-SO^−^, Prdx2-SO^−^, Prdx1-SS, Prdx2-SS and Trx1-SS decrease to half their values adjacent to the membrane 0.35 μm, 0.57 μm, 1.2 μm, 0.65 μm, 1.4 μm and 1.1 μm away, respectively (Fig. 3B). (Due to the uncertainties about the diffusion constants of the proteins and other factors highlighted in SI2, the absolute values presented in this section are merely indicative. The qualitative relationships are robust, though.) The localization of these Prdx oxidized forms agrees with earlier predictions from a simpler model that considered only a single peroxiredoxin [6]. The present model reveals, additionally, a stronger localization of Prdx1-SO^−^ relative to Prdx2-SO^−^ (Fig. 3D), which is due to the ≈20-fold faster resolution step of the former species. Also as a result of Prdx2's slower resolution step, the concentration of Prdx2-SO^−^ in the proximal regions is 2.7-fold that of Prdx1-SO^−^, despite the total concentration of Prdx2 being just 27 % of Prdx1's, and the Prdx2-SO^−^/Prdx1-SO^−^ concentrations ratio increases to ≈400-fold 5 μm away (Fig. 3D, inset). Prdx1-SS is also more localized than Prdx2-SS. This primarily reflects the stronger localization of the former's precursor species, and is just slightly enhanced by the faster reduction of Prdx1-SS by Trx1 relative to Prdx2-SS. Despite this faster reduction, the proximal concentration of Prdx2-SS is just 45 % of Prdx1-SS's, though distally Prdx2-SS is 65.-fold more concentrated than Prdx1-SS (Fig. 3D, inset). In turn, the localization of Trx1-SS (Fig. 3E) primarily reflects the distribution of Prdx1-SS and fast reduction via TrxR. In contrast to the other oxidized peroxiredoxin forms, Prdx1-SO_2_^−^ and Prdx2-SO_2_^−^ are distributed nearly uniformly throughout the aqueous space (Fig. 3B). Unlike the other reduced forms of Prdx1, Prdx2 and Trx1, Prdx1_2_-S^−^ is strongly localized to the H_2_O_2_ sources (Fig. 3C inset).

**Fig. 4:**
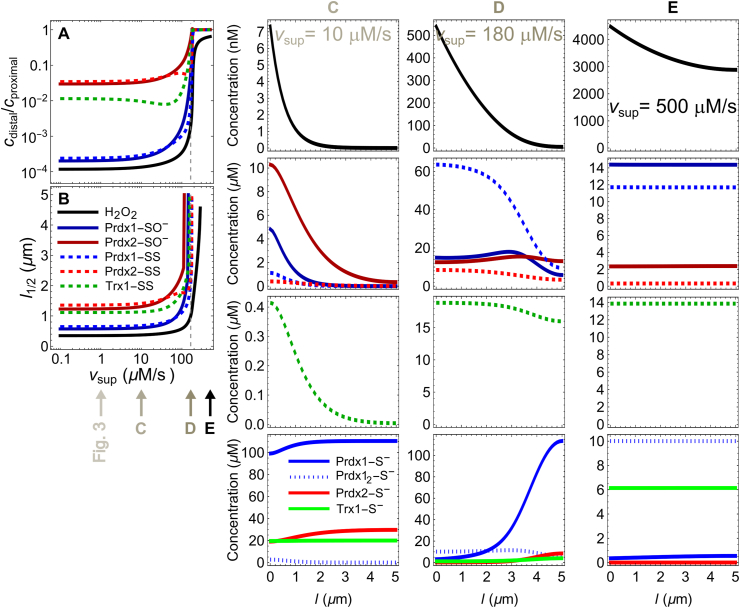
**Dependence of the concentration gradients of H**_**2**_**O**_**2**_**, Prdx and Trx1 species on v**_**sup.**_ (A) Amplitude of the gradients, evaluated as the ratio between the concentration at the source-proximal regions and that at the distal region. The vertical gray dashed line marks V_Max_(TrxR). (B) Distance from the membrane at which the concentrations decrease by 50 %. (B, C, D) Spatial concentration profiles at the *v*_sup_ values indicated by the arrows under panel B. The color/dashing code is the same as in Fig. 3. Note the different concentration scales in the various panels. (For interpretation of the references to color in this figure legend, the reader is referred to the Web version of this article.)

The amplitudes (Fig. 4A) and length scales (Fig. 4B) of the concentration gradients remain almost unchanged over *v*_sup_ values up to one order of magnitude lower than the system's maximal rate of Prdx-SS reduction (Fig. 4C), which in this case is approximately *V*_Max_. But as *v*_sup_ further approaches this capacity, the gradients of the oxidized species become progressively shallower, and those of the reduced species become steeper (Fig. 4A,C,D). At *v*_sup_ values very near *V*_Max_, the concentrations of Prdx1/2-SO^−^ are highest between the proximal and distal regions (Fig. 4D). At even higher v_sup_, the gradients of the Prdx and Trx1 species vanish (Fig. 4A,E). The considered *k*_Alt_ = 100 s^−1^ alternative sink makes the H_2_O_2_ concentration decrease by just 33 % from the proximal to the distal region under these conditions (Fig. 4A,E).

### 3.3 Saturation of the capacity of the Trx1/TrxR system to reduce Prdx disulfides determines the breakdown of localization

**Fig. 5:**
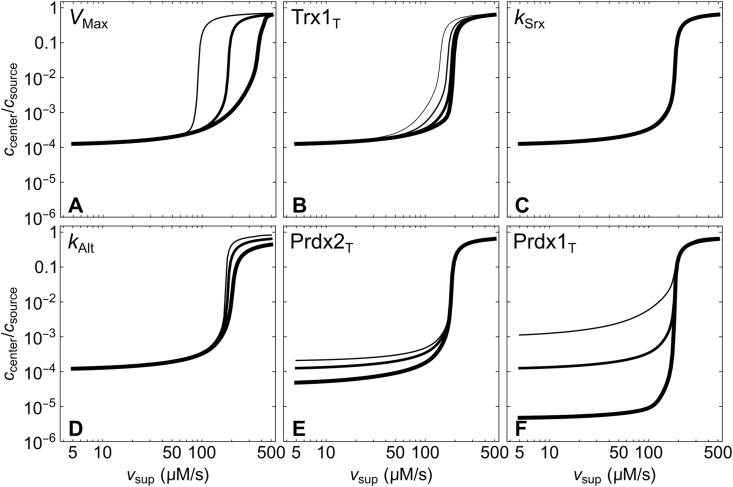
**Effect of the protein concentrations and enzyme activities on the breakdown of the H**_**2**_**O**_**2**_**concentration gradient.** The plots show the H_2_O_2_ gradient amplitudes as a function of the H_2_O_2_ supply rate for 0.5- (thin lines), 1- (medium lines) and 2-fold (thick lines) the reference value of each parameter. In the case of Trx1_T_, the thinnest of the four lines shows the effect of decreasing this parameter to 0.25-fold its reference value. Note the logarithmic scales.

Fig. 5 shows the influence of the various enzyme activities and concentrations on the breakdown of the H_2_O_2_ concentration gradient. For the reference conditions, the main determinant of the maximal *v*_sup_ that cells can sustain while keeping a strong H_2_O_2_ concentration gradient (*v*_sup,__crit_) is the maximal rate of Prdx-SS reduction (Fig. 5A). Here, this rate approximately matches the TrxR activity (*V*_Max_), but Trx1 depletion from the cytosol may further decrease *v*_sup,__crit_ (Fig. 5B). In turn, the total concentrations of Prdx control the amplitude (Fig. 5E and F) and length scale of the H_2_O_2_ gradients, but have almost no influence on *v*_sup,__crit_. The comparatively modest activity of alternative H_2_O_2_ sinks has a marginal influence on the H_2_O_2_ gradients at *v*_sup_ < *v*_sup,__crit_ but it determines the amplitude of the more modest H_2_O_2_ gradients at higher *v*_sup_ (Fig. 5D). Finally, the Srx activity virtually does not influence the H_2_O_2_ gradients (Fig. 5C).

Effects of these enzyme activities and protein concentrations on the gradients of the oxidized Prdx and Trx1 species follow similar trends (Fig. S1). Of note, the Trx1-SS concentration gradient attains its maximal amplitude at intermediate *v*_sup_ values below *v*_sup,_ _crit_ (Fig. S1, last row).

### 3.4 Cytosol's redox polarity increases as H_2_O_2_ supply rates approach the critical value

**Fig. 6:**
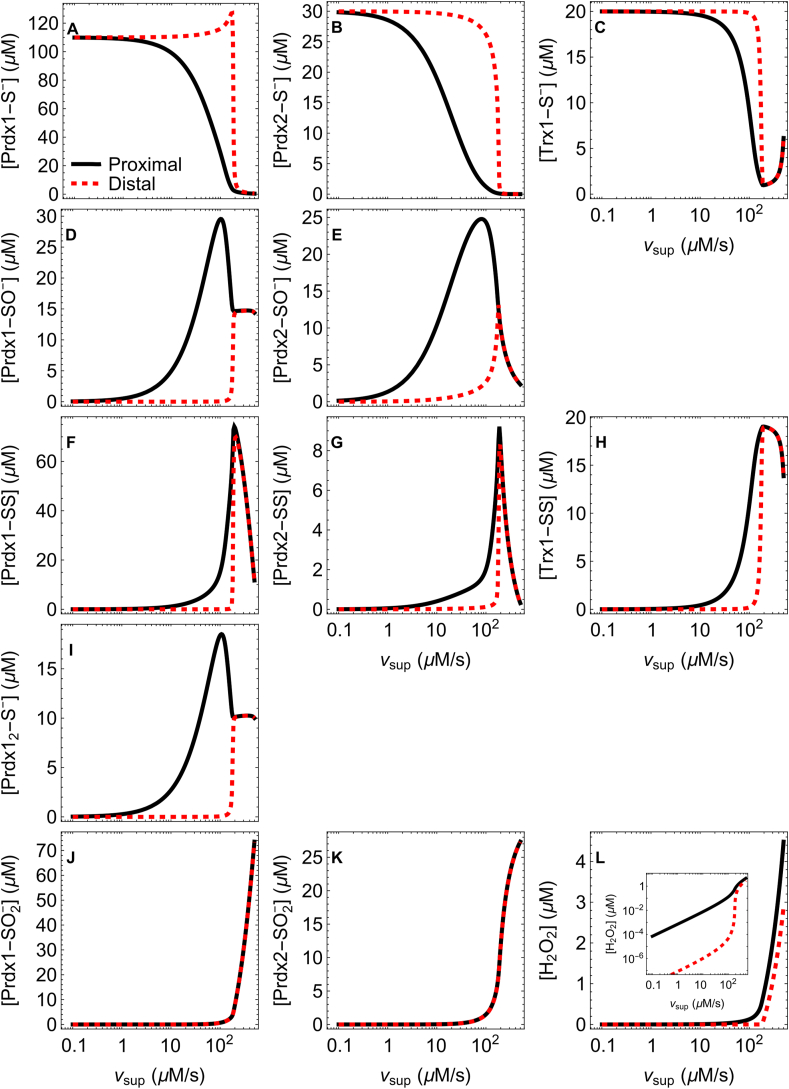
**Steady-state concentrations nearest (black) and farthest (dashed red) the H**_**2**_**O**_**2**_**sources, as a function of the H**_**2**_**O**_**2**_**supply rate.** Note the logarithmic *v*_sup_ scale. The inset in panel L shows the concentrations in logarithmic scale. (For interpretation of the references to color in this figure legend, the reader is referred to the Web version of this article.)

The steady state response of the various species to the H_2_O_2_ supply rate (Fig. 6) is another important consideration for redox signaling. Near the H_2_O_2_ sources the Prdx1, Prdx2 and Trx1 pools remain largely reduced over *v*_sup_ values up to the low μM s^−1^ range (Fig. 6A,B,C,black lines). The Prdx2-S^−^ pool starts being significantly depleted in these locations for *v*_sup_ > 5 μM s^−1^, as the slow resolution step begins to be limiting and Prdx2 accumulates mostly as Prdx2-SO^−^ (Fig. 6E, black line). In turn, the Prdx1-S^−^ and Trx1-S^−^ pools only begin to be significantly depleted at higher *v*_sup_ (>15–20 μM s^−1^) (Fig. 6A,C, black lines), by virtue of the higher resolution rate constant of Prdx1-S^−^ and of the high TrxR activity catalyzing Trx1-SS reduction, respectively. As *v*_sup_ increases, both peroxiredoxins accumulate predominantly in sulfenic form (Fig. 6D and E, black lines). However, as *v*_sup_ further approaches *v*_sup,__crit_ the disulfide forms gain prominence as a consequence of the local depletion of the Trx1-S^−^ pool (Fig. 6F,G,H black lines). At *v*_sup_ in the range of ½- to 2-fold *v*_sup,__crit_, a large fraction Prdx1 locally accumulates in the still poorly characterized reduced but presumably peroxidatically inactive [45] “Prdx1_2_-S^−^“ form (Fig. 6I).

Away from the H_2_O_2_ sources, substantial depletion of the Prdx-S^−^ and Trx1-S^−^ pools occurs only at higher *v*_sup_, approaching the considered 180 μM s^−1^ TrxR activity, but is much more abrupt (Fig. 6A,B,C, dashed red lines). The Prdx1-S^−^ concentration even increases over this *v*_sup_ range, for reasons that will be addressed in the next section. The oxidized forms of both Prdx and of Trx1 only attain μM local concentrations at *v*_sup_ values very close to *v*_sup,__crit_ (Fig. 6D–H, dashed red lines). Therefore, as *v*_sup_ increases up to *v*_sup,__crit_ the redox polarity of the cytosol, as expressed by spatial differences in the availability of reducing equivalents in the Prdx and Trx pools, increases. It then collapses as *v*_sup_ surpasses *v*_sup,__crit_.

At H_2_O_2_ supply rates beyond *v*_sup,__crit_, μM H_2_O_2_ concentrations invade the cytosol (Fig. 6L), and the cytosolic concentration gradients of all the Prdx and Trx species vanish. Concurrently, a large and increasing fraction of both peroxiredoxins accumulates in sulfinic form, throughout the cytosol, decreasing the oxidative load on Trx1 (Fig. 6C, H, J, K).

Considering that Prdx1_2_-S^−^ is decameric (Fig. S2) or that it is instantaneously converted to (active) Prdx1-S^−^ (Fig. S3) does not significantly influence the results above.

In erythrocytes [79] and in some arterial cells [17], Prdx2 is the dominant cytosolic 2-Cys Prdx. In order to examine the consequences of distinct relative abundances of Prdx1 and Prdx2, we repeated the simulations underlying Fig. 6 after swapping the total concentrations of these two proteins (Fig. S4). The spatio-temporal dynamics in this case does not qualitatively differ from that in cells where Prdx1 is the dominant cytosolic 2-Cys Prdx, but it does differ in the following important quantitative aspects. The Trx1-S^−^ pool does not get as strongly depleted as *v*_sup_ reaches *v*_sup,__crit_ (Fig. S4C), because a larger fraction of the Prdx pool accumulates in sulfinic form and the Prdx disulfides do not accumulate as much (compare Figs. S4F,G,J,K to the corresponding panels in Fig. 6, black lines). The distal Prdx1/2-S^−^ pools are depleted more gradually, and Prdx2-SO^−^ and Prdx2-SS accumulate to μM concentrations as *v*_sup_ increases (compare Figs. S4A,B,E,G to the corresponding panels in Fig. 6, dashed red lines). These outcomes are mainly a consequence of the longer lifetime of Prdx2-SO^−^ relative to Prdx1-SO^−^, allowing the former to penetrate farther from the H_2_O_2_ sources.

### 3.5 The lower mobility of prdx decamers relative to dimers helps protect distal regions from H_2_O_2_

**Fig. 7:**
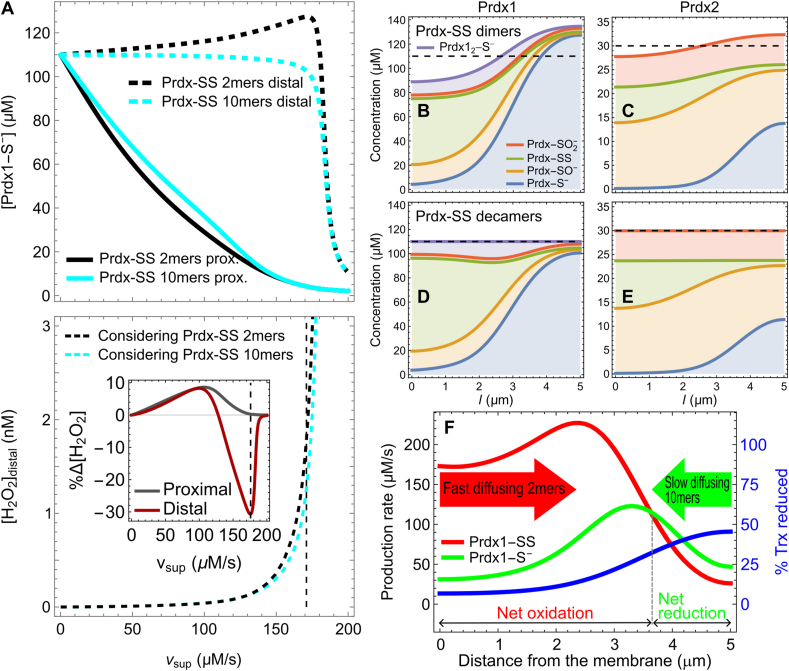
**H**_**2**_**O**_**2**_-**induced Prdx-S**^**−**^**accumulation at distal sites.** (A) Influence of the H_2_O_2_ supply rate on the Prdx1-S^−^ (top panel) and H_2_O_2_ (bottom) concentrations at the most distal (dashed lines) and proximal (solid lines in top panel) sites from the H_2_O_2_ sources, considering Prdx1-SS diffusing as dimers (black lines) or as decamers (cyan lines). The inset in the bottom panel shows the percent difference in H_2_O_2_ concentrations at the proximal (gray) and distal (dark red) sites for Prdx1-SS diffusing as dimers *vs.* diffusing as decamers. The vertical dashed lines mark the *v*_sup_ value causing the greatest percent H_2_O_2_ concentration decrease at the distal site. Distribution of Prdx1 (B, D) and Prdx2 (C, E) species considering Prdx-SS diffusing as dimers (B, C) or decamers (D, E), with *v*_sup_ = 171 μM s^−1^, which yields near maximal distal Prdx1-S^−^ accumulation. The dashed black lines mark the average total Prdx concentration. Note the overall accumulation of Prdx1 and Prdx2 towards distal sites in (B, C). (F) Prdx1-S^−^ (green) and Prdx1-SS (red) production rates and percentage of Trx1 as Trx1-S^−^ (blue, axis at the right side) as function of the distance from the H_2_O_2_ sources for *v*_sup_ = 171 μM s^−1^ with Prdx1-SS diffusing as dimers. Note the excess Prdx1-SS production proximal from the sources and excess Prdx1-S^−^ production distal from the sources. (For interpretation of the references to color in this figure legend, the reader is referred to the Web version of this article.)

The source-distal concentration of Prdx1-S^−^ actually *increases* with the H_2_O_2_ supply rate up to *v*_sup,__crit_ (Fig. 7A, top panel black dashed line). Near *v*_sup,__crit_, the distal Prdx1-S^−^ concentration is ~ 16 % higher than in the absence of a H_2_O_2_ supply. Moreover, the overall pools of both Prdx1 and Prdx2 accumulate distally at *v*_sup_ just below *v*_sup,__crit_ (Fig. 7B and C). These surprising phenomena are due to the interplay between the redox polarity of the environment and the increased mobility of the Prdx-SS dimers relative to the Prdx-S^−^ decamers. More concretely (Fig. 7F), as *v*_sup_ increases and a Trx1 redox gradient develops, Prdx-SS dimers originate mainly near the H_2_O_2_ sources whereas Prdx-S^−^ decamers are regenerated mainly far from the sources, where Trx1-S^−^ remains abundant. Because the large toroidal decamers diffuse more slowly than the dimers, they are more likely to accumulate near their production sites. As a consequence, the overall Prdx pool also accumulates distally. In agreement with this explanation, these phenomena do not occur when the peroxiredoxin disulfide forms are assumed to have the same diffusion constant as the other peroxiredoxin forms (Fig. 7A,D,E). The longer lifetime of Prdx2-SO^−^ relative to Prdx1-SO^−^ attenuates these phenomena in the former Prdx by flattening the Prdx2-SS concentration gradient (Fig. 7C).

As a consequence of the phenomena described in the previous paragraph, at a *v*_sup_ that approaches *v*_sup,__crit_, the distal H_2_O_2_ concentration is up to 31 % lower than it would otherwise be (Fig. 7A, inset in bottom panel), and the mean H_2_O_2_ concentration over the cytosol is also slightly lower (Fig. S5).

Considering that Prdx1_2_-S^−^ is decameric (Fig. S6) or that it is instantaneously converted to (active) Prdx1-S^−^ (Fig. S7) does not significantly influence the results above. In cells such as human hepatocytes that have substantially lower TrxR activity the phenomena described in this section occur at substantially lower *v*_sup_, and are even more pronounced (Fig. S8).

## 4 Discussion

### 4.1 Redox microdomains have a layered architecture around localized H_2_O_2_ sources

We drew on the recent experimental determination of the differential kinetic parameters for the redox cycles of human Prdx1 and Prdx2 (Fig. 1, Table 1) to explore the compositional architecture and dynamics of redox microdomains. The considered concentrations/activities of Prdx, Trx1, TrxR, sulfiredoxin and alternative H_2_O_2_ sinks are typical of human cell lines, and their quantitative relationships are such as to yield these cells’ stereotypical response to H_2_O_2_ as discussed in Ref. [24]. For computational tractability, we considered a geometry where H_2_O_2_ is uniformly supplied to the cytosol from two parallel membranes. However, the qualitative results are expected to apply as well to other geometries where H_2_O_2_ is supplied at discrete sources, such as from endosomes [37], mitochondria [40,80], the endoplasmic reticulum [37,81,82] or peroxisomes [83].

The results show that for H_2_O_2_ supply rates corresponding to basal conditions to moderate stress (SI1) steep concentration gradients of H_2_O_2_ and of the oxidized forms of Prdx1, Prdx2 and Trx1 readily establish once H_2_O_2_ is locally supplied to the cytosol, and reach a steady state in a few seconds (Fig. 2). Unlike the gradients of the other oxidized species, those of the Prdx sulfinic species virtually vanish at steady state (Fig. 2J and K), due to these forms’ slow turnover.

As a consequence of Prdx1-SO^−^‘s faster resolution step, the steady state concentrations of Prdx1-SO^−^ and Prdx1-SS decline by 50 % of their maxima at approximately half the distances from H_2_O_2_ sources relative to their Prdx2 counterparts (Fig. 3B). The Prdx disulfides decay by 50 % just slightly farther from the H_2_O_2_ sources than the respective precursor sulfenates, despite the former diffusing faster (as dimers) than the latter. This happens due to the fast reduction of the disulfide forms by Trx1, resulting in half-lives of just 10–70 ms. In turn, Trx1-SS decays to half of its maximum concentration at about the same distance from the H_2_O_2_ supply sites as Prdx2-SO^−^, for the reference TrxR activity. Importantly, all these concentrations decrease approximately exponentially with distance from the H_2_O_2_ sources, which has the following two relevant implications for redox signaling. Firstly, farther than ≈3 μm from H_2_O_2_ sources, the relevant oxidized Prdx species may already be too rarefied (nM concentrations, Fig. 3D inset) to oxidize redox targets in the few-minutes time frame observed in experiments (e.g., [[11], [84], [85], [86]]). [Note that the fastest known oxidation of other proteins by Prdx-SS, that of Trx1-S^−^, has rate constants in the 10^6^ M^−1^s^−1^ range (Table 1), and no comparably fast oxidations by Prdx-SO^−^ are known.] Therefore, these species can't directly actuate targets anchored at distant sites. For diffusible targets, most of the Prdx-mediated signal transduction must occur proximal to H_2_O_2_ sources, and the resulting target modifications must be stable enough to reach possibly distant actuation points such as gene promoters. Secondly, because the concentrations of oxidized species decrease nearly exponentially with the distance from the H_2_O_2_ sources, the seemingly small differences in the gradients' length scales in Fig. 4B translate into orders-of-magnitude differences in gradient amplitude and concentration a few μm away (Fig. 3D inset, Fig. 4A). Thus, for the reference conditions and a 1 μM s^−1^ H_2_O_2_ supply rate, the Prdx2-SO^−^/Prdx1-SO^−^ concentration ratio grows from 2.7 to ≈400 when comparing proximal regions to distal ones 5 μm away (Fig. 3D); and whilst at the proximal regions the concentration of Prdx1-SS is 2.2-fold higher than Prdx2-SS's, it is 65-fold *lower* at the distal regions. Therefore, for similarly reactive targets, Prdx1-SO^−^ and Prdx1-SS should act closer to the H_2_O_2_ sources than the corresponding Prdx2 forms. This remains true over a wide range of H_2_O_2_ supply rates, almost up to those causing the collapse of the gradients (Fig. 4).

Nevertheless, the above-characterized localization may not fully avoid interference between the potentially thousands of discrete sites releasing H_2_O_2_ to the cytosol (in mitochondria, peroxisomes, endocytic vesicles, cell membrane, etc.). Whether mediation of redox relays by site-targeted protein scaffolds, such as recently described for the Prdx2-STAT3 relay [87], can avoid such interference is currently under investigation.

It must be noted that the absolute values of the gradient length scales and amplitudes above are merely indicative, for the reasons discussed in SI2. However, the conclusions about the relative localization of Prdx1 *versus* Prdx2 forms are robust.

### 4.2 The activity of the peroxiredoxin disulfide reduction system determines the H_2_O_2_ supply rates at which the gradients collapse

The amplitudes and length scales of the concentration gradients described above are almost invariant over aggregate H_2_O_2_ supply rates (*v*_sup_) up to one order of magnitude lower than the maximal rate at which the Trx/TrxR system can reduce Prdx-SS (Fig. 4). Under these conditions, the H_2_O_2_ supply rate *at each discrete source* determines the local concentrations of H_2_O_2_, Prdx and Trx species, as long as the sources are separated enough to avoid mutual interference. At higher *v*_sup_ values, the Prdx-S^−^ pools become gradually more depleted. As a consequence, the H_2_O_2_'s half-life increases, allowing these molecules to diffuse farther and thus flatten the gradient. The gradients of the resulting oxidized species can be no steeper than those of the respective precursors, and therefore become shallower as well. When the *aggregated v*_sup_ (*i.e.*, the sum of the supply rates at all sources) surpasses the cytosol's Prdx-SS reduction capacity, the peroxiredoxins become completely oxidized. Because the activity of the alternative sinks is too low to sustain a significant H_2_O_2_ gradient, all the gradients then collapse and H_2_O_2_ invades the cytosol at μM concentrations. Of all the enzyme activities and protein concentrations considered in the model, only the TrxR activity, and to a lesser extent the total Trx1 concentration, control the value of *v*_sup_ at which the gradients collapse (Fig. 5). In turn, the Prdx concentration is the main variable controlling the amplitude and length scale of the H_2_O_2_ gradient, the most abundant Prdx having the greatest influence.

Although the TrxR activity hardly influences the H_2_O_2_ concentrations at low *v*_sup_, it gains a substantial influence as *v*_sup_ approaches TrxR's apparent maximal rate (Fig. 5A). This happens for the following reason. At these *v*_sup_ the TrxR becomes progressively less able to keep up with the flux of Trx1-SS generated by the reduction of Prdx1/2-SS. As a consequence, the Trx1-S^−^ pool becomes depleted, and Prdx1/2-SS reduction becomes the rate-limiting step in the Prdx catalytic cycle. This causes these Prdx to accumulate as Prdx1/2-SS, depleting the Prdx1/2-S^−^ pools. Under these conditions, the abundance of the latter pools becomes strongly dependent on TrxR activity, and therefore so does the H_2_O_2_ concentration. These theoretical results are in full agreement with and explain the recent experimental observations of Hoehne *et al.* [40] that cells' TrxR content strongly influences their responses to μM-range H_2_O_2_ boluses and their cytosolic H_2_O_2_ concentration gradients.

These results also help explaining why, in contrast to other redox relays [87,88], the Prdx1-ASK1 redox relay does not need the help of a scaffold protein to operate [89]. In this redox relay, Prdx1-SS oxidizes ASK1's C250 which then forms disulfide-bonded multimers that become active as a mitogen-activated protein kinase kinase kinase, eventually triggering apoptosis [[89], [90], [91], [92]]. However, Trx1-S^−^ binds ASK1, hindering access to C250, thereby preventing its oxidation [93,94], and also reducing the disulfide-bonded multimers, inactivating them [91]. Therefore, the Prdx1-SS – ASK1 relay can only operate and effectively oxidize ASK1 when the Trx1-S^−^ pool is strongly depleted. Under these conditions, the gradients collapse and ≈75 μM Prdx1-SS becomes available throughout the cytosol (Fig. 6F). At this concentration, Prdx1-SS can oxidize ASK1 in seconds, given the 3 × 10^4^ M^−1^s^−1^ rate constant [89] for this interaction.

The computational predictions for H_2_O_2_ supply rates in excess of *v*_sup,__crit_ are also in keeping with experimental observations of the cellular responses to H_2_O_2_ stress. As *v*_sup_ further increases, a large and increasing fraction of both peroxiredoxins accumulates in sulfinic form throughout the cytosol, decreasing the oxidative load on Trx1 (Fig. 6C, H, I, J). A similar phenomenon has been experimentally documented for the peroxiredoxin Tpx1 in the fission yeast *Schizosaccharomyces pombe* [95]. The relief of the oxidative load on thioredoxin caused by Tpx1 hyperoxidation proved essential to allow the former protein to assist the repair of oxidative damage and ensure cell survival following exposure to high H_2_O_2_ concentrations [95]. Moreover, because Prdx1/2-SO_2_^−^ can function as holdases that prevent the aggregation of unfolding client proteins, their accumulation to high concentrations may favor cell survival [96,97]. However, it is unclear if such high *v*_sup_ are ever attained in human physiology.

The breakdown of localization with increasing *v*_sup_ is associated with a smooth threshold-like response. This supports the predictions [24] for cells with a similar protein composition, based on a coarse-grained homogeneous-system kinetic model that did not explicitly consider the distinct 2-Cys peroxiredoxins in the cytosol of human cells. In turn, the model in Ref. [6] predicted that the breakdown of spatial localization is associated to a *v*_sup_ range where two stable steady states coexist, leading to a hysteretic (*i.e.*, trigger-like) response. This is likely a consequence of the approximations underlying the latter model, though it may occur in some cell types (further discussion in SI3).

### 4.3 Localized H_2_O_2_ supply and fast kinetics relative to diffusion polarize the cytosolic redox environment

Perhaps the most striking feature from the analysis of the steady state response to localized H_2_O_2_ supply (Fig. 6) is the development, with increasing *v*_sup_, of a stark redox polarization between H_2_O_2_-source-proximal and distal regions. In proximal regions, the local Prdx1/2-S^−^ and Trx1-S^−^ pools are gradually depleted as *v*_sup_ increases beyond ≈5 μM s^−1^, and Prdx and Trx1 disulfides, as well as Prdx sulfenates, accumulate to high-μM concentrations. These local conditions are suitable for the operation of Prdx- and/or Trx1-mediated redox relays. In contrast, in distal regions the Prdx and Trx1 pools remain highly reduced up to *v*_sup_ values approaching the cytosolic capacity to reduce Prdx1/2-SS, and only then abruptly collapse. These local reducing conditions minimize oxidative damage to local cellular components, but are unsuitable for the operation of the above-mentioned redox relays due to the very low local concentrations of the oxidized Prdx and Trx1 forms. These conclusions hold as well if Prdx1_2_-S^−^ is decameric or instantaneously converts to Prdx1-S^−^ (Figs. S3 and 4).

### 4.4 Peroxiredoxins' redox-oligomeric coupling enhances the cytosol's redox polarization

The just-discussed redox polarization is a consequence of H_2_O_2_ and oxidized Prdx and Trx1 forms in this system — save for the Prdx sulfinic forms — having short lifetimes that do not allow them to diffuse far from the H_2_O_2_ sources. A coupling between Prdx's redox and oligomeric states is not necessary for such a redox polarization, but further enhances it, as explained below.

Although the extent to which Prdx1/2-SS dissociates into dimers *in vivo* remains uncertain, even a partial dissociation has the following two surprising consequences: (i) the concentration of Prdx1-S^−^ at distal sites even *increases* with increasing *v*_sup_, and (ii) the Prdx pool partially migrates away from H_2_O_2_ sources towards distal regions (Fig. 7). The emergence of a sustained intracellular concentration gradient of a total protein pool has been observed [98] and theoretically explained [99] in the context of kinase-phosphatase systems. For this phenomenon to occur, the alternative forms of the protein must have distinct diffusion coefficients, and at least one of the reactions interconverting them must be spatially localized [99].

In the present context, these phenomena significantly decrease H_2_O_2_ concentrations at distal sites (Fig. 7A, bottom panel) and slightly decrease the total H_2_O_2_ concentration in the cytosol (Fig. S5), with the greatest effect for stresses that bring the Prdx-S^−^ pool to the verge of collapse. In turn, their effect on the source-proximal H_2_O_2_ concentration under the same circumstances is minimal (Fig. 7A, gray line in the inset of the bottom panel). Therefore, under oxidative stress a higher mobility of the Prdx-SS relative to other Prdx redox states allocates the dwindling Prdx-S^−^ pool to where it can have the greatest protective effect. It is uncertain if the high *v*_sup_ at which these phenomena are predicted for the tumor cell line simulated in this work are relevant for human physiology. However, these phenomena are predicted to occur at substantially lower *v*_sup_ in differentiated cells, which tend to have much lower TrxR activities (Fig. S8). Other factors that may modulate them and/or affect the attending estimates are discussed in SI4.

### 4.5 Concluding remarks

In summary, this work highlights that redox microdomains are not just sites of H_2_O_2_ accumulation. They are also enriched in oxidized Prdx and Trx species that are relevant for signal transduction. The relative abundances of these species change, and their concentrations exponentially decay, with increasing distance from the H_2_O_2_ sources. As a consequence, a strong redox polarization of the cytosol between H_2_O_2_-source-proximal and distal regions emerges. The same likely happens in the mitochondrial matrix, and in the lumens of other organelles that contain abundant peroxiredoxins and peroxidases. Despite the small dimensions in some directions, the eccentric and convoluted shapes of some of these organelles prompt the generation of substantial concentration gradients if the reactive species are supplied at discrete and widely separated sites.

A coupling between redox and oligomeric states of the 2-Cys Prdxs, even if partial, further enhances that redox polarization by promoting the accumulation of the active reduced forms distally from H_2_O_2_ sources under oxidative stress.

In the cytosol, the sulfenic and disulfide Prdx1 forms are more localized than the corresponding Prdx2 species. However, the oxidation of regulatory targets in redox relays that operate in the absence of oxidative stress by any of these peroxiredoxins must occur primarily in the vicinity of H_2_O_2_ sources. This is unlike redox relays that operate under stress conditions where the gradients collapse, such as that of Prdx1-ASK1.

The main factor determining what H_2_O_2_ supply rates cells tolerate until redox gradients collapse is the capacity of the peroxiredoxin disulfide reduction system, which in most human cell lines is determined by the TrxR activity. At H_2_O_2_ supply rates approaching this capacity, the concentrations of H_2_O_2_ and of oxidized Prdx and Trx1 species become very sensitive to the TrxR activity, in contrast to what is observed at H_2_O_2_ supply rates up to the low μM s^−1^.

Altogether, these results highlight the importance of protein diffusion limitations and modulation for redox signaling and antioxidant protection.

## Funding

Work financed by the European Regional Development Fund, through COMPETE2020-Operational Program for Competitiveness and Internationalization, and Portuguese funds via FCT-Fundação para a Ciência e a Tecnologia, under projects UIDB/04539/2020, UIDP/04539/2020, LA/P/0058/2020, UIDB/00324/2020, UIDP/00313/2020, UIDB/04564/2020 and UIDP/04564/2020. Matthew Griffith has been supported by the 10.13039/501100000835University of Bath and a NERC GW4+ Doctoral Training Partnership studentship from the UK 10.13039/501100000270Natural Environment Research Council (grant no. NE/L002434/1).

## Competing financial interests

The authors declare no competing financial interests.

## Declaration of competing interest

The authors declare that they have no known competing financial interests or personal relationships that could have appeared to influence the work reported in this paper.

## Data Availability

Data will be made available on request.
