# Supplementary material for: The architecture of redox microdomains: Cascading gradients and peroxiredoxins’ redox-oligomeric coupling integrate redox signaling and antioxidant protection
Source: Redox Biol. 2023 Dec 21;69:103000. doi: 10.1016/j.redox.2023.103000 (PMC10829873; doi:10.1016/j.redox.2023.103000)
Supplement: Multimedia component 1 [file mmc1.pdf]

Supplementary Information for
The architecture of redox microdomains: cascading gradients and peroxiredoxins' redox-oligomeric coupling integrate redox signaling and antioxidant protection

Matthew Griffith[1,2], Adérito Araújo[3*], Rui Travasso[4**], Armindo Salvador[1,5,6***]

[1] CNC - Centre for Neuroscience Cell Biology, University of Coimbra, UC-Biotech, Parque Tecnológico de Cantanhede, Núcleo 4, Lote 8, 3060-197 Cantanhede, Portugal
[2] Department of Mathematical Sciences, University of Bath, Claverton Down, Bath BA2 7AY, UK
[3] CMUC, Department of Mathematics, University of Coimbra, Largo D. Dinis, 3004-143 Coimbra, Portugal
[4] CFisUC, Department of Physics, University of Coimbra, Coimbra, Rua Larga, 3004-516 Coimbra, Portugal
[5] Coimbra Chemistry Center - Institute of Molecular Sciences (CQC-IMS), University of Coimbra, Rua Larga, 3004-535 Coimbra, Portugal
[6] Institute for Interdisciplinary Research, University of Coimbra, Casa Costa Alemão, Rua Dom Francisco de Lemos, 3030-789 Coimbra, Portugal

Contents

1. Estimation of H_2O_2 supply rates under various conditions 2
2. Factors affecting the estimates of gradient length scales and amplitudes 4
3. Factors determining a hysteretic response to H_2O_2 supply 5
4. Factors affecting the total protein concentration gradients and attending phenomena 5
5. References 5
6. Supplementary Figures 9

1. Estimation of H_2O_2 supply rates under various conditions

Below we draw on published data about superoxide and H_2O_2 production (v_{sup}) in various cell types and conditions to assess what H_2O_2 supply rates are pertinent *in vivo*.

Mitochondria are widely believed to be major $O_2^{\bullet-}$ and H_2O_2 suppliers to the cytosol, but the supply rates *in vivo* under physiological conditions can only be indirectly estimated. Gonçalves et al. [1] determined $O_2^{\bullet-}/H_2O_2$ release rates from isolated Wistar rat muscle mitochondria incubated in media mimicking the cytoplasmic substrate and effector mix of skeletal muscle during rest and during exercise. Values ranged from 100 (for intense aerobic exercise) to 336 pmol H_2O_2 equivalents / min / mg mitochondrial protein (for rest).[1] Considering a 0.2 g/mL protein concentration and that the total mitochondrial volume is $\approx$10% of cytosol's, this range translates into v_{sup}= 37. – 124. nM s^{-1}. This estimate is very contingent on the medium composition including all the physiologically relevant cytosolic substrates and effectors in the right proportions. Therefore, estimates based on *in cellula* determinations should in principle be more reliable.

Fang et al. [2] determined $O_2^{\bullet-}/H_2O_2$ release rates of various types of human, rat and mouse cultured cells, as well as the relative contributions of NADPH oxidases (NOX), sites I_Q and III_{Qo} of the mitochondrial respiratory chain and other unspecified (presumably cytosolic) sources. These release rates ranged from 2.9 pmol H_2O_2 equivalents / min / mg protein, for HeLa cells, to 84. pmol H_2O_2 equivalents / min / mg protein, for AML12 mouse liver cells. These values convert to 9.7 nM s^{-1} - 280. nM s^{-1}, referred to cell volume. The relative contributions from the various sources were remarkably consistent, despite type (primary, immortalized, cancerous), tissue (cervix, lung, bone, neuron, skin, heart, liver) and species (human, mouse, rat) differences. NOXs were the main contributors (42-77%), and other non-mitochondrial sources contributed relatively little (up to 23%). Importantly, these rates and contributions reflect a balance between production and clearance rates. The cytosolic peroxiredoxins should consume an overwhelming fraction of the H_2O_2 released to the cytosol under these conditions. Therefore, the large contribution of NOXs is quite likely due to several of these releasing $O_2^{\bullet-}/H_2O_2$ directly to the extracellular medium, and the release rate attributed to them should approximately represent this direct extracellular release. We can draw on previous estimates [3] of cytosolic 2-Cys peroxiredoxin concentration (65. μM) and effective permeation rate constant (12. s^{-1}) for HeLa cells to estimate the fraction of H_2O_2 supplied to the cytosol that is released to the extracellular environment, and from there estimate v_{sup}. Considering a 100 $\mu M^{-1}s^{-1}$ rate constant for the reaction of the 2-Cys peroxiredoxins with H_2O_2 the released fraction is (12. s^{-1})/((100 $\mu M^{-1}s^{-1}$)$\times$(65. μM) + (12. s^{-1}))= 0.0018, meaning that about 1 every 560 H_2O_2 molecules supplied to the cytosol is released to the extracellular medium. Discounting the 65% of H_2O_2 equivalents attributed to NOXs in these cells, one obtains a (1 - 0.65)$\times$(9.7 nM s^{-1})= 3.4 nM s^{-1} release rate from intracellular sources. From this value, considering that the cytosol accounts for 78% of the volume of a HeLa cell [4] and only 70% of it is occupied by water [5], one infers v_{sup}= (3.4 nM s^{-1})/ (0.0018$\times$0.7$\times$0.78)= 3.5 μM s^{-1}. This value quite likely overestimates the physiological basal rate, for the following three reasons. Firstly, because the rupture of even a small fraction of the cells during handling can greatly increase the H_2O_2 release rate, given that the intact permeability barrier retains 99.8% of the H_2O_2 molecules. Secondly, because diffusion limitations and partial Prdx inhibition decrease the effective

---

[1] The term "H_2O_2 equivalents" here denotes the amount of H_2O_2 detected after dismutation of all the $O_2^{\bullet-}$ also released.

clearance rate. Thirdly, the much lower pO_2 *in vivo* should result in a somewhat lower v_{sup} than that inferred from the experiments conducted at atmospheric pressure [6]. However, it is unlikely that these factors together explain the large discrepancy with respect to the estimate in the previous paragraph.

Countering the notion that cell rupture seriously inflated the determined release rate, note that for a 3.4 nM s^{-1} release rate to obtain the H_2O_2 concentration near the cellular membrane has to be (3.4 nM s^{-1})/(12. s^{-1})= 0.28 nM. This value is one order of magnitude *lower* than the mean cytosolic H_2O_2 concentration determined for undisturbed K562 cells [7] (but still a reasonable agreement given the approximations involved and the distinct cell types and experimental conditions).

The v_{sup} estimates obtained for the other two human epithelial tumor cell lines considered in both refs. [2,3] — A549 and U-2 OS — are 2.6 and 12. μM s^{-1}, respectively, assuming that the membranes of these cells are as permeable to H_2O_2 and HeLa's. Whether the substantially higher H_2O_2 release rates of H9c2 rat heart myoblasts and AML12 mouse liver cells [2] reflect higher mitochondrial release (eventually reflecting the higher metabolic rate of these small mammals) and/or lower cytosolic clearance capacity is unknown.

Peroxisomes are another potentially relevant H_2O_2 source. Specially so in hepatocytes, where they are most abundant. A recent kinetic model of peroxisomal H_2O_2 metabolism in rat hepatocytes [8] predicts a 0.9 μM s^{-1} H_2O_2 release rate to the cytosol under basal conditions, which might increase up to 15. μM s^{-1} upon catalase inactivation.

A recent model [9] estimated that the H_2O_2 influx through the apical face of endothelial cells in normal-functioning vasculature does not exceed ≈50 nM s^{-1} (referred to the cytosolic volume), which is modest in comparison to cell-endogenous values.

Altogether, the estimates above suggest that basal v_{sup} in human cells are in the 100s of nM s^{-1} to low μM s^{-1} range. In turn, the following address stress situations.

Phagosomes may leak H_2O_2 to the cytosol of neutrophils at high rates. A kinetic model of the reactions of $O_2^{\bullet-}$ and myeloperoxidase in neutrophil phagosomes [10] estimates a 2 μM steady state concentration of H_2O_2 within these particles, and a 70 s^{-1} effective permeability rate constant. This yields a (2 μM)×(70 s^{-1})= 140 μM s^{-1} permeation rate, referred to phagosome volume. Since a phagosome and a neutrophil have 1.5 μm and 8.5 μm [11] diameters, respectively, the volume ratio is $(1.5/8.5)^3$= 0.0055. Considering that each neutrophil carries 20 phagosomes and that the cytosolic water volume corresponds to ≈50% of the neutrophil volume, one finds v_{sup}(phagosomes)= 20×0.0055×(140 μM s^{-1})/0.5= 31. μM s^{-1}.

Extracellular sources may contribute substantially to v_{sup} under some circumstances. In zebrafish, tail wounding stimulated H_2O_2 production that was sufficient to totally oxidize the Prdx pool of the cells near wound margins, reaching a ≈5 μM concentration [12]. If one assumes that these cells are as large and have membranes as H_2O_2-permeable as typical human cells, yielding effective influx rate constants in the 10 – 20 s^{-1} range [3,13], this extracellular concentration corresponds to a v_{sup}= 50 – 100 μM s^{-1}. However, subsequent apoptosis of these cells appears to be intrinsic to and necessary for wound repair [14]. Likewise, exposure of Jurkat T cells to >5 μM H_2O_2 steady states, which in these cells correspond to v_{sup}> 26 μM s^{-1} [3], already causes significant apoptosis [15,16], and H_2O_2 concentrations that are insufficient to totally oxidize the Prdx pool may already induce apoptosis of HeLa cells [17]. Altogether, these findings

strongly suggest that v_{sup} values in the high tens of μM s^{-1} represent overt stress, at least for some cell types.

Nevertheless, Fichman *et al.* [18] have recently shown that localized injury of tissues with mM H_2O_2 or other agents triggers a wave of high H_2O_2 concentrations that propagates over macroscopic distances. It is hard to see how such a H_2O_2 signal could propagate over such a long range in a tissue constituted by H_2O_2-permeant cells without a sufficient v_{sup} being generated to extensively oxidizing the cytosolic redox pools. Yet, there was no evidence that this wave had a toxic effect, which suggests that cells in their natural environment may be more resistant to H_2O_2 than cells in culture.

2. Factors affecting the estimates of gradient length scales and amplitudes

The absolute values of the gradient length scales and amplitudes presented in this work are merely indicative, for the following two reasons. Firstly, because we had to estimate the diffusion constants of peroxiredoxins and thioredoxins based on those determined for other similarly sized proteins diffusing in the cytosol. Such estimates are quite uncertain, because other molecular properties and the cell's physiological state may strongly influence protein diffusion [19]. For instance, interactions with large proteins and fixed cell structures may strongly delay diffusion [20], as can a decrease in free cytosolic solvent volume caused by an osmotic shock [21,22]. There is also considerable cell-to-cell variation in diffusion coefficients [22]. Nevertheless, the square root dependence of the gradient length scales on the diffusion constants partly mitigates the effect of these uncertainties on the results. Moreover, Prdx1 and Prdx2 have very similar sizes and biophysical properties, and the *relative* length scales of distinct redox forms of the same Prdx are essentially determined by their precursor-product relationships. Therefore, the above-mentioned uncertainties about the diffusion coefficients are very unlikely to upend the conclusions about the relative localization of Prdx1 *versus* Prdx2 forms.

Secondly, although the length scale of the H_2O_2 gradient is virtually insensitive to the diffusion constants of the proteins in most conditions, H_2O_2 *production* in the cytosol may be delocalized to some extent. Particularly relevant here is the possibility that most of the H_2O_2 is supplied through dismutation of superoxide released to the cytosol [9]. In this case, if superoxide diffuses substantially before undergoing dismutation, much of the H_2O_2 will be produced far away from the superoxide sources, blunting the H_2O_2 gradient and those of the Prdx and Trx forms. However, although superoxide dismutase 1 (SOD1) is just 10% to 20% as abundant as Prdx1+Prdx2 in the cytosol of human cells [23], SODs react >10-fold faster with superoxide as these Prdxs do with H_2O_2 [24], and their reactivity is not limited by the availability of co-substrates. Therefore, superoxide molecules should diffuse no farther than ≈0.5 μM on average until undergoing dismutation. As a consequence, this indirect H_2O_2 supply should not severely compromise the spatial localization of H_2O_2 and of the Prdx and Trx species. Indeed, no H_2O_2 is detected by very sensitive probes targeted to the cell membrane or to the nucleus when superoxide production by complex III of the mitochondrial electron transport chain is stimulated by antimycin treatment of HEK293 cells [25]. This occurs even though mitochondria can be in close proximity to these cellular structures. Thus, most of the superoxide is dismutated within mitochondria, and/or the cytosolic SOD activity in these cells suffices to keep H_2O_2 very localized.

3. Factors determining a hysteretic response to H_2O_2 supply

As the subsequent analysis in ref. [3] revealed, the hysteretic behavior in ref. [26] ensues from two approximations made in ref. [26]. Namely, that (i) the neglect of alternative H_2O_2 sinks, which is pertinent for endothelial cells, and (ii) the neglect of Trx1 oxidation, which is pertinent for the low v_{sup} that were the main focus in ref. [26] but not at the higher v_{sup} where the breakdown occurs. Although a hysteretic response can occur under some circumstances in the absence of those approximations and may be biologically relevant [3], we are unaware of any experimental data to either support or disprove its occurrence *in vivo*.

4. Factors affecting the total protein concentration gradients and attending phenomena

These phenomena occur as well if $Prdx1_2\text{-}S^-$ is decameric or instantaneously converts to $Prdx1\text{-}S^-$ (Figures S5,6). A partial dissociation of Prdx1/2-SS into dimers [27–31] attenuates them. But on the other hand, the model is prone to underestimate their extent for the following three reasons. Firstly, the approach we used (see "Model and parameter estimates" section of the main text) likely overestimates the diffusivity of Prdx decamers, because it treats them as spherical proteins with a radius commensurate to their MW and thus neglects the higher hydrodynamic radius of these toroidal molecules. Secondly, the numerous interactions of both Prdx and Trx1 with other proteins may further slow their diffusion in the cytosol, thereby enhancing all the gradients. Thirdly, several known features that the current model neglects for the sake of simplicity or because they remain poorly characterized may further amplify the described phenomena. For example, the intradimer cooperativity of Prdx [32], and the inhibition of Trx1-SS's reduction *via* TrxR [33] caused by the oxidation of Trx1's regulatory thiols by Prdx-SS [34] or by Trx1 dimerization [35]. In contrast, the stabilization of Prdx1-SS decamers through the formation of dimer-to-dimer disulfide bridges via the oxidation of Cys83 [31] should attenuate the aforementioned effects (Figure 7A, D, E). In turn, glutathionylation of Cys83 destabilizes the decamers [29]. How these modifications of Prdx1 Cys83 are regulated *in vivo* is unknown, but studies with a homo-FRET probe indicate that in cells, Prdx1 dissociates into dimers when oxidized to Prdx1-SS [36]. The interplay between all these factors in integrating redox signaling and antioxidant protection is a fascinating topic for further theoretical and experimental analysis.

5. References

[1] R.L.S. Goncalves, C.L. Quinlan, I. V Perevoshchikova, M. Hey-Mogensen, M.D. Brand, Sites of Superoxide and Hydrogen Peroxide Production by Muscle Mitochondria Assessed ex Vivo under Conditions Mimicking Rest and Exercise, J. Biol. Chem. 290 (2015) 209–227. doi:10.1074/jbc.M114.619072.

[2] J. Fang, H.S. Wong, M.D. Brand, Production of superoxide and hydrogen peroxide in the mitochondrial matrix is dominated by site IQ of complex I in diverse cell lines, Redox Biol. 37 (2020) 101722. doi:10.1016/J.REDOX.2020.101722.

[3] G. Selvaggio, P.M.B.M. Coelho, A. Salvador, Mapping the phenotypic repertoire of the cytoplasmic 2-Cys peroxiredoxin – thioredoxin system. 1. Understanding commonalities and differences among cell types, Redox Biol. 15 (2018) 297–315. doi:10.1016/j.redox.2017.12.008.

[4] A. Fujioka, K. Terai, R.E. Itoh, K. Aoki, T. Nakamura, S. Kuroda, E. Nishida, M. Matsuda, Dynamics

of the Ras/ERK MAPK Cascade as Monitored by Fluorescent Probes, J. Biol. Chem. 281 (2006) 8917–8926. doi:10.1074/jbc.M509344200.

[5] R. Milo, R. Phillips, Cell Biology by the Numbers, 1st ed., Taylor & Francis, New York, 2016.

[6] D.L. Hoffman, J.D. Salter, P.S. Brookes, Response of mitochondrial reactive oxygen species generation to steady-state oxygen tension: implications for hypoxic cell signaling, Am. J. Physiol. Circ. Physiol. 292 (2007) H101–H108. doi:10.1152/ajpheart.00699.2006.

[7] O. Lyublinskaya, F. Antunes, Measuring intracellular concentration of hydrogen peroxide with the use of genetically encoded H2O2 biosensor HyPer, Redox Biol. 24 (2019) 101200. doi:10.1016/J.REDOX.2019.101200.

[8] M.J. Ferreira, T.A. Rodrigues, A.G. Pedrosa, L. Gales, A. Salvador, T. Francisco, J.E. Azevedo, The mammalian peroxisomal membrane is permeable to both GSH and GSSG – Implications for intraperoxisomal redox homeostasis, Redox Biol. 63 (2023) 102764. doi:10.1016/j.redox.2023.102764.

[9] T. Sousa, M. Gouveia, R.D.M. Travasso, A. Salvador, How abundant are superoxide and hydrogen peroxide in the vasculature lumen, how far can they reach?, Redox Biol. 58 (2022) 102527. doi:10.1016/j.redox.2022.102527.

[10] C.C. Winterbourn, M.B. Hampton, J.H. Livesey, A.J. Kettle, Modeling the Reactions of Superoxide and Myeloperoxidase in the Neutrophil Phagosome, J. Biol. Chem. 281 (2006) 39860–39869. doi:10.1074/jbc.M605898200.

[11] H. Ting-Beall, D. Needham, R. Hochmuth, Volume and Osmotic Properties of Human Neutrophils, Blood. 81 (1993) 2774–2780. doi:10.1182/BLOOD.V81.10.2774.2774.

[12] M. Jelcic, B. Enyedi, J.B. Xavier, P. Niethammer, Image-Based Measurement of H2O2 Reaction-Diffusion in Wounded Zebrafish Larvae, Biophys. J. 112 (2017) 2011–2018. doi:10.1016/j.bpj.2017.03.021.

[13] M.N. Möller, E. Cuevasanta, F. Orrico, A.C. Lopez, L. Thomson, A. Denicola, Diffusion and transport of reactive species across cell membranes, Adv. Exp. Med. Biol. 1127 (2019) 3–19. doi:10.1007/978-3-030-11488-6_1.

[14] C. Rampon, C. Gauron, F. Meda, M. Volovitch, S. Vriz, Adenosine enhances progenitor cell recruitment and nerve growth via its A2B receptor during adult fin regeneration, Purinergic Signal. 10 (2014) 595–602. doi:10.1007/S11302-014-9420-9/FIGURES/6.

[15] F. Antunes, E. Cadenas, Cellular titration of apoptosis with steady state concentrations of H2O2: submicromolar levels of H2O2 induce apoptosis through Fenton chemistry independent of the cellular thiol state, Free Radic. Biol. Med. 30 (2001) 1008–1018. doi:10.1016/S0891-5849(01)00493-2.

[16] F. Antunes, E. Cadenas, U.T. Brunk, Apoptosis induced by exposure to a low steady-state concentration of H2O2 is a consequence of lysosomal rupture, Biochem. J. 356 (2001) 549–555. doi:10.1042/bj3560549.

[17] B.K. Huang, H.D. Sikes, Quantifying intracellular hydrogen peroxide perturbations in terms of concentration, Redox Biol. 2 (2014) 955–962. doi:10.1016/j.redox.2014.08.001.

[18] Y. Fichman, L. Rowland, M.J. Oliver, R. Mittler, ROS are evolutionary conserved cell-to-cell stress signals, Proc. Natl. Acad. Sci. 120 (2023) e2305496120. doi:10.1073/pnas.2305496120.

[19] P.E. Schavemaker, A.J. Boersma, B. Poolman, How Important Is Protein Diffusion in Prokaryotes?, Front. Mol. Biosci. 5 (2018) 395408. doi:10.3389/fmolb.2018.00093.

[20] P.E. Schavemaker, W.M. Śmigiel, B. Poolman, Ribosome surface properties may impose limits on the nature of the cytoplasmic proteome, Elife. 6 (2017). doi:10.7554/eLife.30084.

[21] M.C. Konopka, K.A. Sochacki, B.P. Bratton, I.A. Shkel, M.T. Record, J.C. Weisshaar, Cytoplasmic Protein Mobility in Osmotically Stressed Escherichia coli, J. Bacteriol. 191 (2009) 231–237. doi:10.1128/JB.00536-08.

[22] J.T. Mika, P.E. Schavemaker, V. Krasnikov, B. Poolman, Impact of osmotic stress on protein diffusion in <scp> *L* </scp> *actococcus lactis*, Mol. Microbiol. 94 (2014) 857–870. doi:10.1111/mmi.12800.

[23] T. Geiger, A. Wehner, C. Schaab, J. Cox, M. Mann, Comparative Proteomic Analysis of Eleven Common Cell Lines Reveals Ubiquitous but Varying Expression of Most Proteins, Mol. Cell. Proteomics. 11 (2012) M111.014050-M111.014050. doi:10.1074/mcp.M111.014050.

[24] H.J. Forman, I. Fridovich, Superoxide dismutase: A comparison of rate constants, Arch. Biochem. Biophys. 158 (1973) 396–400. doi:10.1016/0003-9861(73)90636-X.

[25] M.N. Hoehne, L.J.H.C. Jacobs, K.J. Lapacz, G. Calabrese, L.M. Murschall, T. Marker, H. Kaul, A. Trifunovic, B. Morgan, M. Fricker, V. V Belousov, J. Riemer, Spatial and temporal control of mitochondrial H2O2 release in intact human cells, EMBO J. 41 (2022) e109169. doi:10.15252/embj.2021109169.

[26] R.D.M. Travasso, F. Sampaio dos Aidos, A. Bayani, P. Abranches, A. Salvador, Localized Redox Relays as a Privileged Mode of Cytoplasmic Hydrogen Peroxide Signaling, Redox Biol. 12 (2017) 233–245. doi:10.1016/j.redox.2017.01.003.

[27] Z.A. Wood, L.B. Poole, R.R. Hantgan, P.A. Karplus, Dimers to Doughnuts: Redox-Sensitive Oligomerization of 2-Cysteine Peroxiredoxins, Biochemistry. 41 (2002) 5493–5504. doi:10.1021/bi012173m.

[28] S. Barranco-Medina, J.-J. Lázaro, K.-J. Dietz, The oligomeric conformation of peroxiredoxins links redox state to function, Febs Lett. 583 (2009) 1809–1816. doi:http://dx.doi.org/10.1016/j.febslet.2009.05.029.

[29] J.W. Park, G. Piszczek, S.G. Rhee, P.B. Chock, Glutathionylation of Peroxiredoxin I Induces Decamer to Dimers Dissociation with Concomitant Loss of Chaperone Activity, Biochemistry. 50 (2011) 3204–3210. doi:10.1021/bi101373h.

[30] R.A. Poynton, A. V Peskin, A.C. Haynes, W.T. Lowther, M.B. Hampton, C.C. Winterbourn, Kinetic analysis of structural influences on the susceptibility of peroxiredoxins 2 and 3 to hyperoxidation, Biochem. J. 473 (2016) 411–421. doi:10.1042/BJ20150572.

[31] W. Lee, K.-S. Choi, J. Riddell, C. Ip, D. Ghosh, J.-H. Park, Y.-M. Park, Human Peroxiredoxin 1 and 2 Are Not Duplicate Proteins, J. Biol. Chem. 282 (2007) 22011–22022. doi:10.1074/jbc.M610330200.

[32] A. V. Peskin, F.C. Meotti, L.F. de Souza, R.F. Anderson, C.C. Winterbourn, A. Salvador, Intra-dimer cooperativity between the active site cysteines during the oxidation of peroxiredoxin 2, Free Radic. Biol. Med. 158 (2020) 115–125. doi:10.1016/j.freeradbiomed.2020.07.007.

[33] W.H. Watson, J. Pohl, W.R. Montfort, O. Stuchlik, M.S. Reed, G. Powis, D.P. Jones, Redox potential of human thioredoxin 1 and identification of a second dithiol/disulfide motif, J. Biol. Chem. 278 (2003) 33408–33415. doi:10.1074/jbc.M211107200.

[34] Y. Du, H. Zhang, X. Zhang, J. Lu, A. Holmgren, Thioredoxin 1 Is Inactivated Due to Oxidation Induced by Peroxiredoxin under Oxidative Stress and Reactivated by the Glutaredoxin System, J. Biol. Chem. 288 (2013) 32241–32247. doi:10.1074/jbc.M113.495150.

[35] C.H. Lillig, A. Holmgren, Thioredoxin and Related Molecules–From Biology to Health and Disease, Antioxid. Redox Signal. 9 (2007) 25–47. doi:10.1089/ars.2007.9.25.

[36] D. Pastor-Flores, D. Talwar, B. Pedre, T.P. Dick, Real-time monitoring of peroxiredoxin oligomerization dynamics in living cells, Proc. Natl. Acad. Sci. (2020) 201915275. doi:10.1073/pnas.1915275117.

6. Supplementary Figures

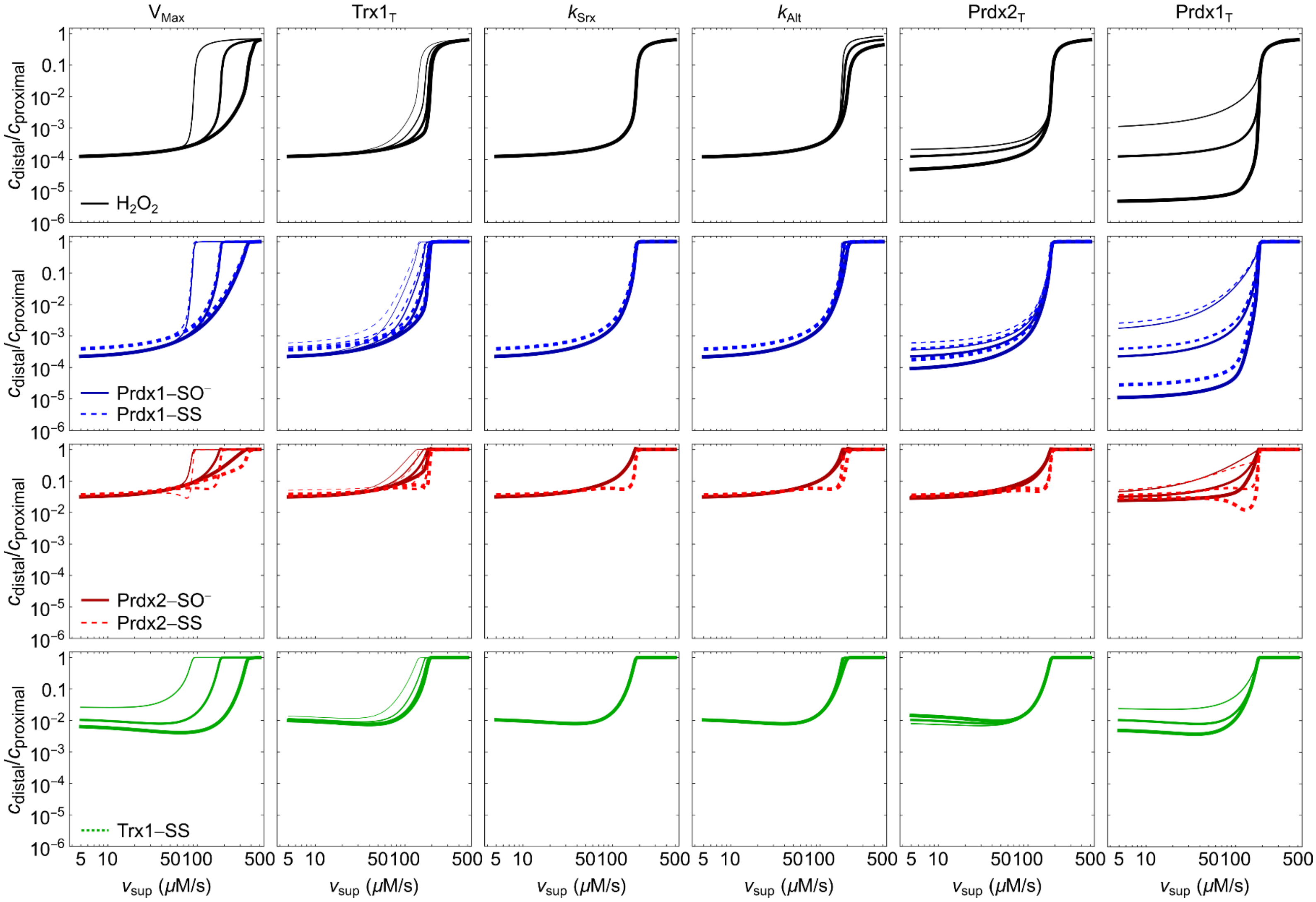

Figure S 1. Effect of the protein concentrations and enzyme activities on the concentration gradients of H_2O_2 and oxidized Prdx and Trx1 species. *The plots show the gradient amplitudes as a function of the H_2O_2 supply rate for 0.5- (thin lines), 1- (medium lines) and 2-fold (thick lines) the reference value of each parameter. In the case of $Trx1_T$, the thinnest of the four lines shows the effect of decreasing this parameter to 0.25-fold its reference value. Note the logarithmic scales.*

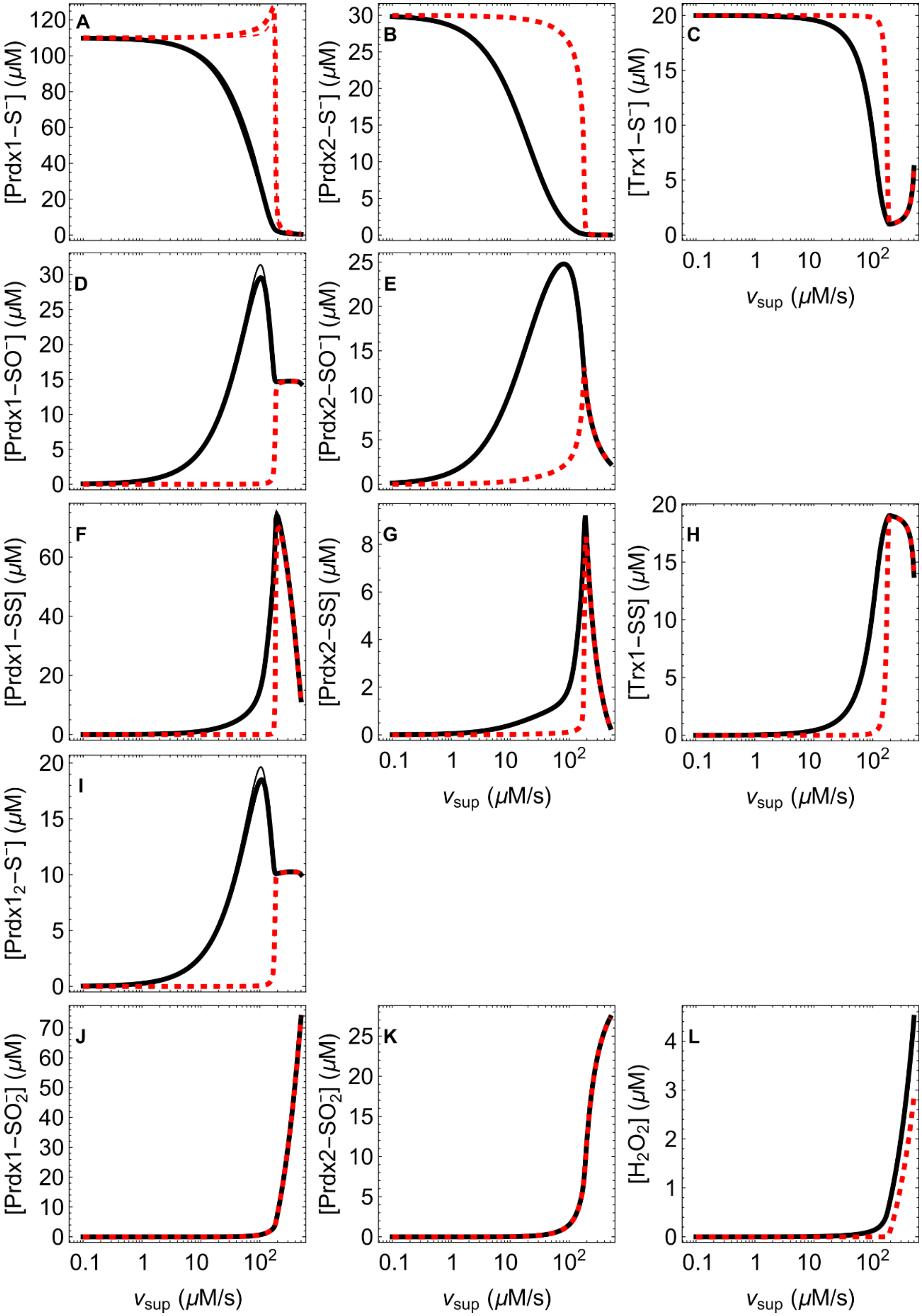

Figure S 2. Steady-state concentrations nearest (black) and farthest (dashed red) from the H_2O_2 source as a function of the H_2O_2 supply rate for a system where the species $Prdx1_2$-S^- diffuses as decamers. *The thin lines show the concentrations obtained in the reference model where $Prdx1_2$-S^- diffuses as dimers. Note the logarithmic v_{sup} scale.*

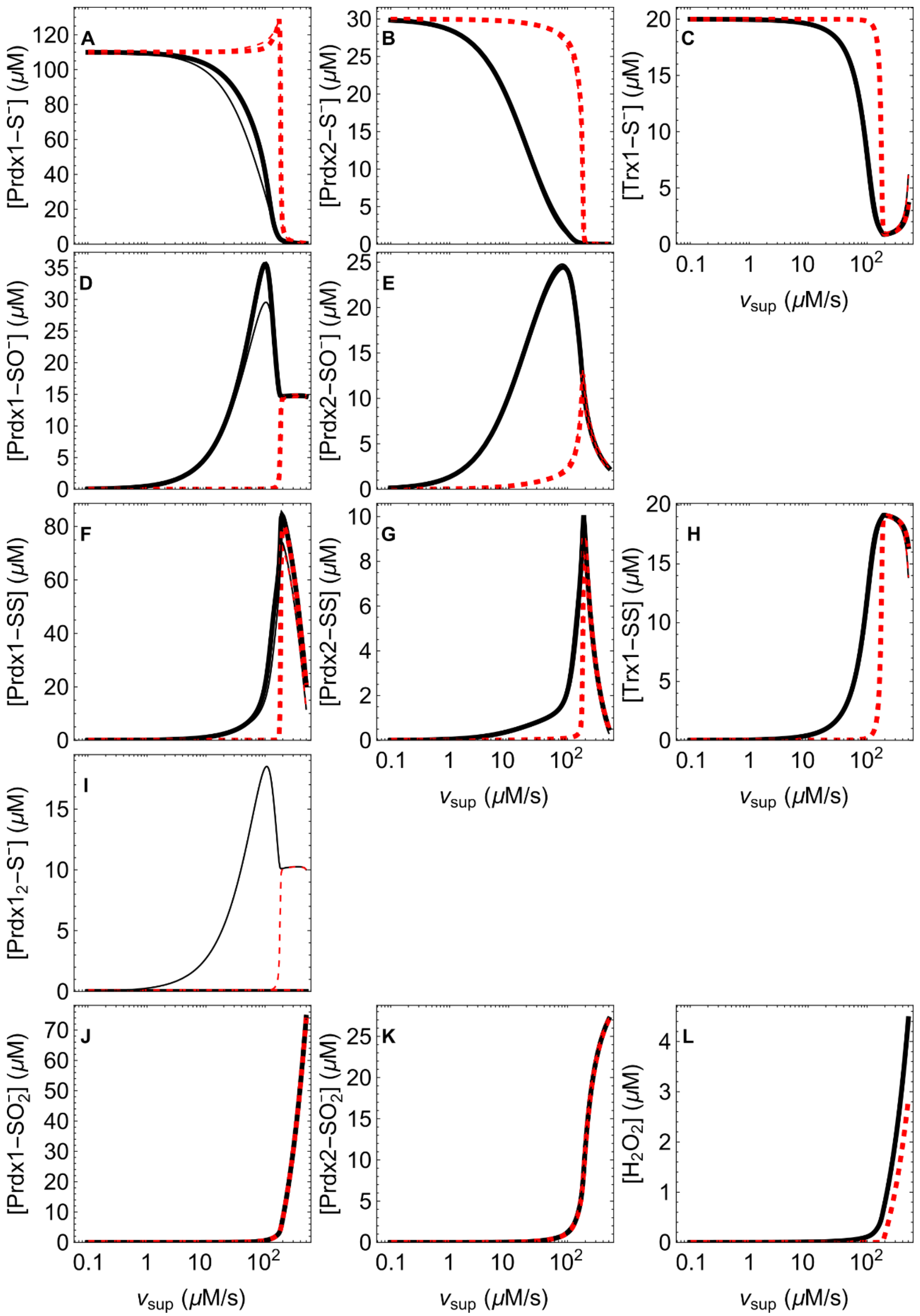

Figure S 3. Steady-state concentrations nearest (black) and farthest (dashed red) from the H_2O_2 source as a function of the H_2O_2 supply rate for a system where conversion of Prdx1$_2$-S$^-$ into Prdx1-S$^-$ is instantaneous. *The thin lines show the concentrations obtained in the reference model. Note the logarithmic v_{sup} scale.*

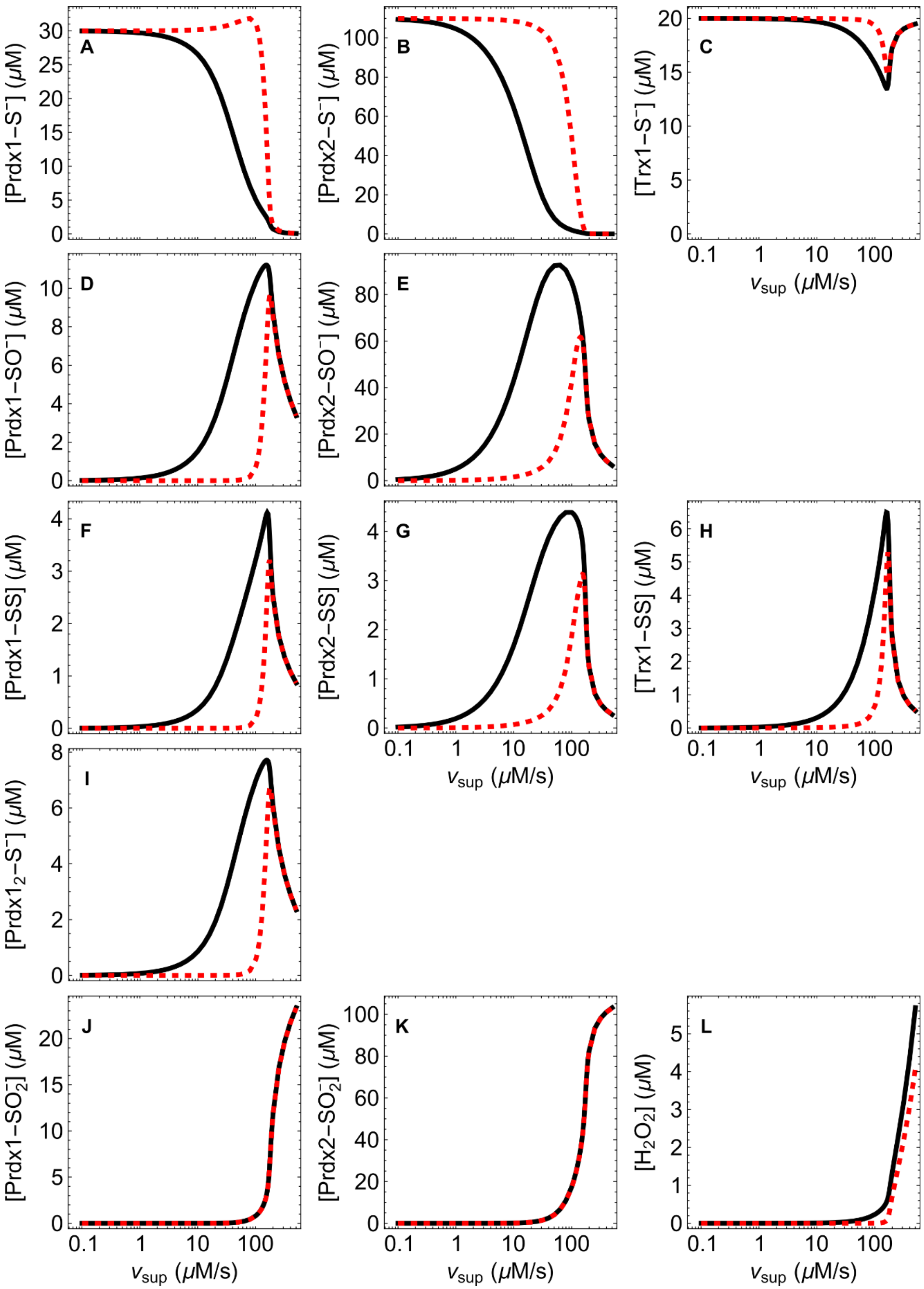

Figure S 4. Steady-state concentrations nearest (black) and farthest (dashed red) from the H_2O_2 source as a function of the H_2O_2 supply rate for a system where Prdx2 is the most abundant 2-Cys Prdx. *$Prdx1_T$= 30 µM, $Prdx2_T$= 110 µM, other parameters as in Table 1. Note the logarithmic v_{sup} scale.*

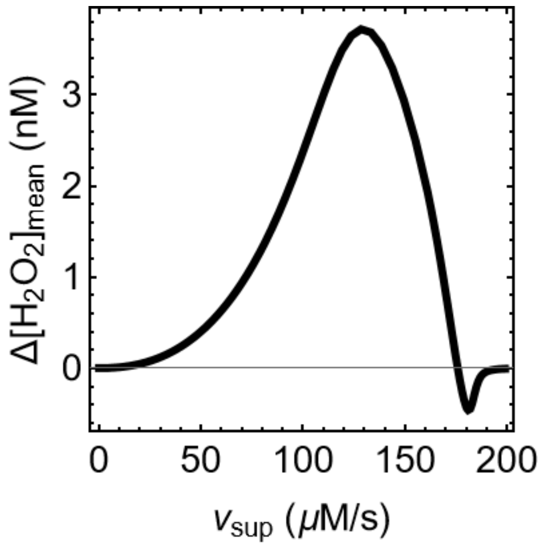

Figure S 5. Difference between mean steady state H_2O_2 concentration over the cytosolic domain obtained considering that Prdx1/2-SS diffuse as dimers and that obtained considering that Prdx1/2-SS diffuse as decamers.

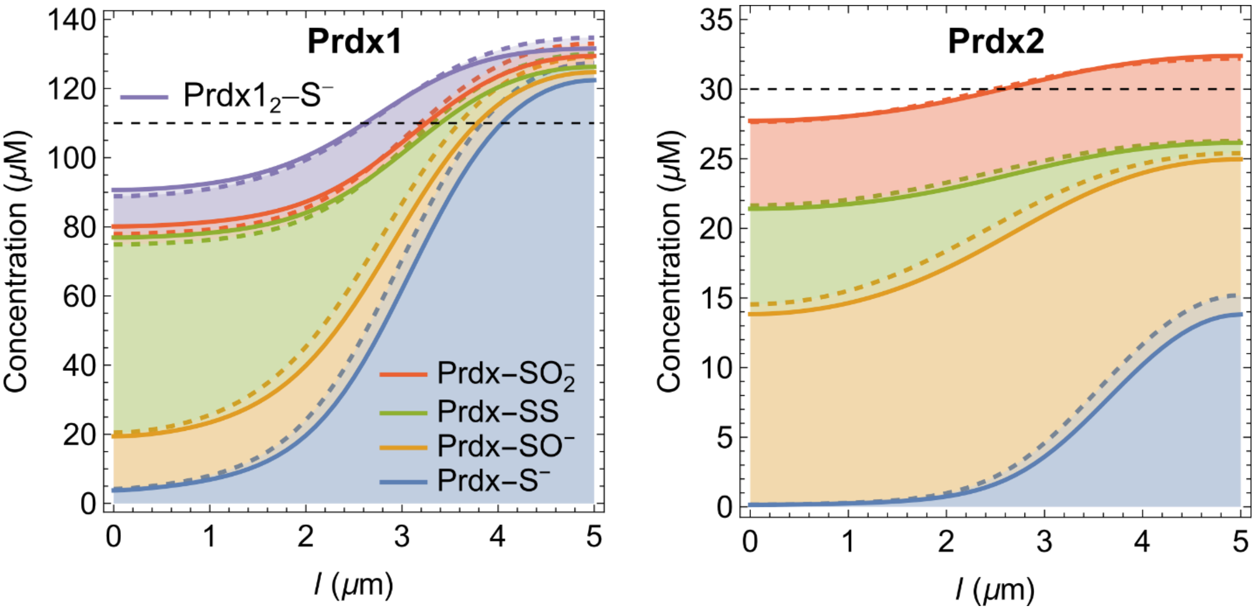

Figure S 6. Distribution of Prdx1 and Prdx2 species considering Prdx-SS diffusing as dimers and Prdx1$_2$-S$^-$ diffusing as decamers (solid lines) or dimers (dashed lines), with v_{sup}= 171 μM s^{-1}, which yields near maximal distal Prdx1-S$^-$ accumulation.

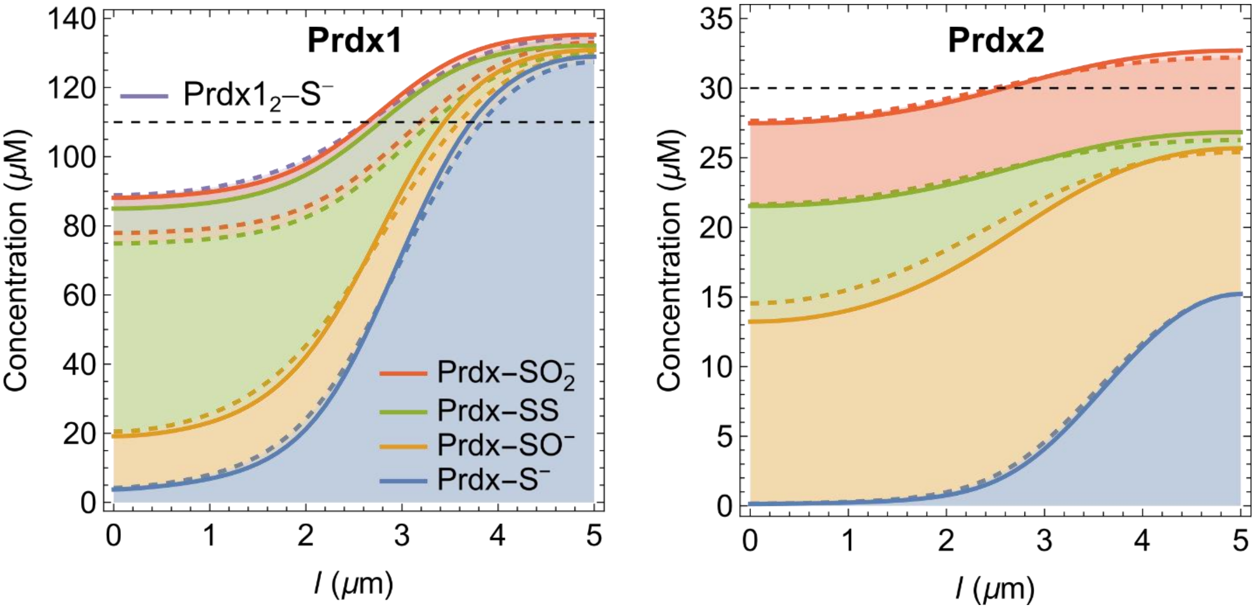

Figure S 7. Distribution of Prdx1 and Prdx2 for a system where Prdx1-SS reduction occurs in a single step (solid lines) or in two steps as in the reference model (dashed lines), with v_{sup}= 171 μM s^{-1}, which yields near maximal distal Prdx1-S$^-$ accumulation.

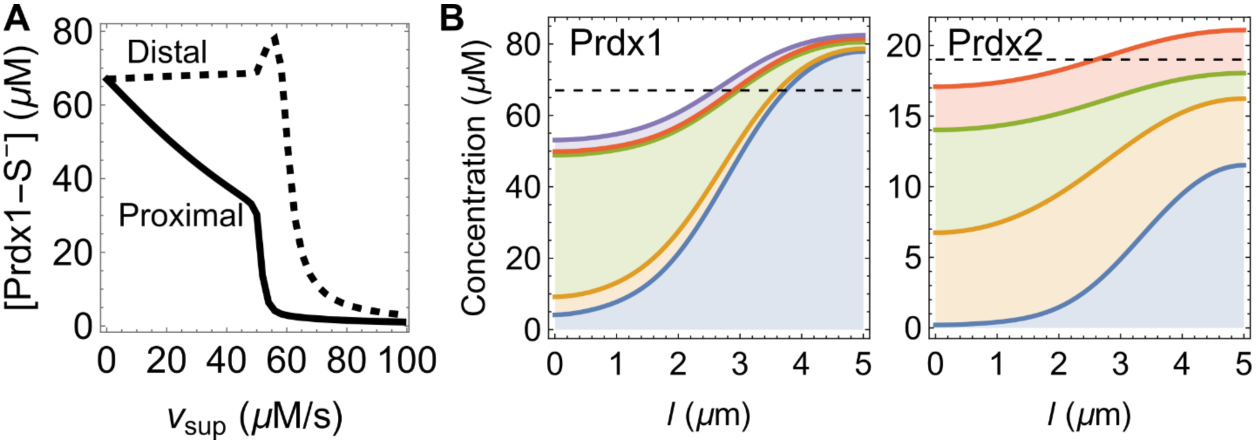

Figure S 8. Response of spatial distribution of Prdx species in human hepatocytes. *(A) Response of the Prdx1-S⁻ concentrations in the proximal and distal regions to H_2O_2 supply rates. (B) Spatial distribution of the Prdx forms at the H_2O_2 supply rate yielding the highest distal Prdx1-S⁻ concentration. Parameters are k_{Alt}= 200 s^{-1}, k_{Srx}= 2.9×10^{-4} s^{-1}, V_{Max}= 50. μM s^{-1}, Prdx1$_T$= 67. μM, Prdx2$_T$= 19. μM, Trx1$_T$= 63. μM. All other parameters are as in Table 1.*
